# An Essential Role of the Arginine Vasotocin System in Mate-Guarding Behaviors in Triadic Relationships of Medaka Fish (*Oryzias latipes*)

**DOI:** 10.1371/journal.pgen.1005009

**Published:** 2015-02-26

**Authors:** Saori Yokoi, Teruhiro Okuyama, Yasuhiro Kamei, Kiyoshi Naruse, Yoshihito Taniguchi, Satoshi Ansai, Masato Kinoshita, Larry J. Young, Nobuaki Takemori, Takeo Kubo, Hideaki Takeuchi

**Affiliations:** 1 Department of Biological Sciences, Graduate School of Science, The University of Tokyo, Tokyo, Japan; 2 Laboratory of Bioresources, National Institute for Basic Biology, Okazaki, Aichi, Japan; 3 Department of Basic Biology, School of Life Science, The Graduate University for Advanced Studies (SOKENDAI), Okazaki, Japan; 4 The Spectrography and Bioimaging Facility, National Institute for Basic Biology, Okazaki, Aichi, Japan; 5 NIBB Center of the Interuniversity Bio-Backup Project, National Institute for Basic Biology, Okazaki, Aichi, Japan; 6 Department of Public Health and Preventive Medicine, Kyorin University, School of Medicine, Mitaka, Tokyo, Japan; 7 Division of Applied Biosciences, Graduate School of Agriculture, Kyoto University, Kyoto, Japan; 8 Center for Translational Social Neuroscience, Department of Psychiatry and Behavioral Sciences, Yerkes National Primate Research Center, Emory University, Atlanta, Georgia, United States of America; 9 Proteo-Science Center, Division of Proteomics Research, Ehime University, Toon City, Ehime, Japan; K.U.Leuven, BELGIUM

## Abstract

To increase individual male fitness, males of various species remain near a (potential) mating partner and repel their rivals (mate-guarding). Mate-guarding is assumed to be mediated by two different types of motivation: sexual motivation toward the opposite sex and competitive motivation toward the same sex. The genetic/molecular mechanisms underlying how mate presence affects male competitive motivation in a triadic relationship has remained largely unknown. Here we showed that male medaka fish prominently exhibit mate-guarding behavior. The presence of a female robustly triggers male-male competition for the female in a triadic relationship (2 males and 1 female). The male-male competition resulted in one male occupying a dominant position near the female while interfering with the other male's approach of the female. Paternity testing revealed that the dominant male had a significantly higher mating success rate than the other male in a triadic relationship. We next generated medaka mutants of arginine-vasotocin (*avt*) and its receptors (*V1a1*, *V1a2*) and revealed that two genes, *avt* and *V1a2*, are required for normal mate-guarding behavior. In addition, behavioral analysis of courtship behaviors in a dyadic relationship and aggressive behaviors within a male group revealed that *avt* mutant males displayed decreased sexual motivation but showed normal aggression. In contrast, heterozygote *V1a2* mutant males displayed decreased aggression, but normal mate-guarding and courtship behavior. Thus, impaired mate-guarding in *avt* and *V1a2* homozygote mutants may be due to the loss of sexual motivation toward the opposite sex, and not to the loss of competitive motivation toward rival males. The different behavioral phenotypes between *avt*, *V1a2* heterozygote, and *V1a2* homozygote mutants suggest that there are redundant systems to activate V1a2 and that endogenous ligands activating the receptor may differ according to the social context.

## Author Summary

Males of various species, including humans, remain near a (potential) mating partner and repel their rival males (mate-guarding). Mate-guarding is mediated by two different types of motivation: sexual motivation toward the opposite sex and competitive motivation toward the same sex. Here we show that the arginine-vasotocin (AVT), a non-mammalian homolog of arginine-vasopressin (AVP), system mediates the molecular mechanisms underlying how mate presence (intersexual relationship) affects male competitive motivation (intrasexual relationship) in mate-guarding (a triadic relationship). We first established a novel behavioral paradigm in which medaka robustly exhibit mate-guarding behavior, that allowed us to study the genetic/molecular mechanisms underlying mate-guarding. Behavioral analysis of courtship behaviors, aggressive behaviors and mate-guarding using medaka mutants in this paradigm suggested that the AVT system is involved in the process in which mate (female) presence drives sexual motivation of males, which may facilitate mate guarding in the triadic relationship. Our study provides genetic evidence that the AVT/AVP system regulates mate-guarding behaviors, suggesting that the role of this system in mate-guarding is evolutionarily conserved from teleosts to mammals.

## Introduction

Male mating strategies are considered to take two major forms: intersexual interaction (female-male interaction) and intrasexual competition (male-male competition) and there is extensive literature focusing on the neural/molecular mechanisms underlying these strategies [1]. In addition to these mating strategies, males of various species, including insects [2, 3], birds [4, 5], mammals [6], primates [7], and humans [8], exhibit mate-guarding behaviors in which they remain near a (potential) mating partner and repel their rival males, which involves both intersexual and intrasexual interactions. Lack of attention to either a mating partner or rival males in mate-guarding would allow the rivals to approach and mate with the partner, known as sneaking [9] and extra-pair copulations [10–12]. In fact, ecological studies indicate that mate-guarding is required to increase individual male fitness in some vertebrates [6, 12].

As mate-guarding in a triadic relationship comprises both inter- and intra-sexual interaction [2–8], mate-guarding is assumed to require two different types of motivation: sexual motivation toward the opposite sex and competitive motivation toward the same sex. Little attention has been paid to the genetic/molecular mechanisms underlying how mate presence (intersexual interaction) affects male competitive motivation (intrasexual interaction) in a triadic relationship, mainly because of the lack of an established behavioral system that robustly elicits this type of complex behavior under laboratory conditions for any genetic model organism. To explore this issue, we focused on medaka fish (*Oryzias latipes*), which is a commonly used model animal in molecular genetics. The medaka mating system involves socially-regulated female preference (intersexual interaction) [13–16] and male-male competition (intrasexual interaction) [17, 18]. Medaka fish exhibit mating behavior every morning, because sexually mature females have a 24-h reproductive cycle. The medaka mating behavior comprises sequential steps, such as male courtship display and synchronized mating [13–16]. Although there are a number of reports on medaka males aggressive behaviors toward other males in multi-male groups [17, 18], there is only one previous report describing the emergence of mate-guarding behavior in a triadic relationship of medaka fish (1 female and 2 males) [19]. This behavior in medaka fish has not been quantitatively investigated, however, because there has been no applicable assay to reliably assess this behavior.

In the present study, to investigate the genetic/molecular mechanisms underlying mate-guarding behavior, we developed a behavioral assay that robustly elicits mate-guarding behavior in medaka. We then examined the possible involvement of arginine-vasotocin (AVT), a non-mammalian homolog of arginine-vasopressin (AVP). In monogamous male prairie voles, the AVP system is involved in mating-induced selective aggression toward a non-mate in a dyad, which may represent mate-guarding [20, 21]. In teleost fish, AVT is implicated in various kinds of social behaviors, such as territorial behavior in a tropical damselfish [22, 23] and pair bonding in a monogamous cichlid fish [24]. In particular, AVT increases both courtship and territorial behaviors of non-territorial males in the bluehead wrasse in the field [25], implying that the AVT system has a key role in the motivation of mating-related behaviors. The possible involvement of the AVT/AVP system in actual mate-guarding in a triadic relationship, however, has not been investigated in these species under laboratory conditions. To evaluate the requirement of the molecular components of the AVT pathways in the regulation of mate-guarding, loss of function analysis using knockout (KO) animals is a valid and feasible method. Here we generated medaka *avt* and V1a type AVT receptor 1 (*V1a1*) and V1a type AVT receptor 2 (*V1a2*) mutants using advanced molecular genetics, such as the TILLING (Targeting Induced Local Lesions IN Genomes) [26, 27] and TALEN (Transcription Activator-Like Effector Nucleases) methods [28, 29], and examined how the AVT pathway is involved in mate-guarding behaviors.

## Results

### Emergence of mate-guarding behavior in a triadic relationship in medaka fish

**Fig 1:**
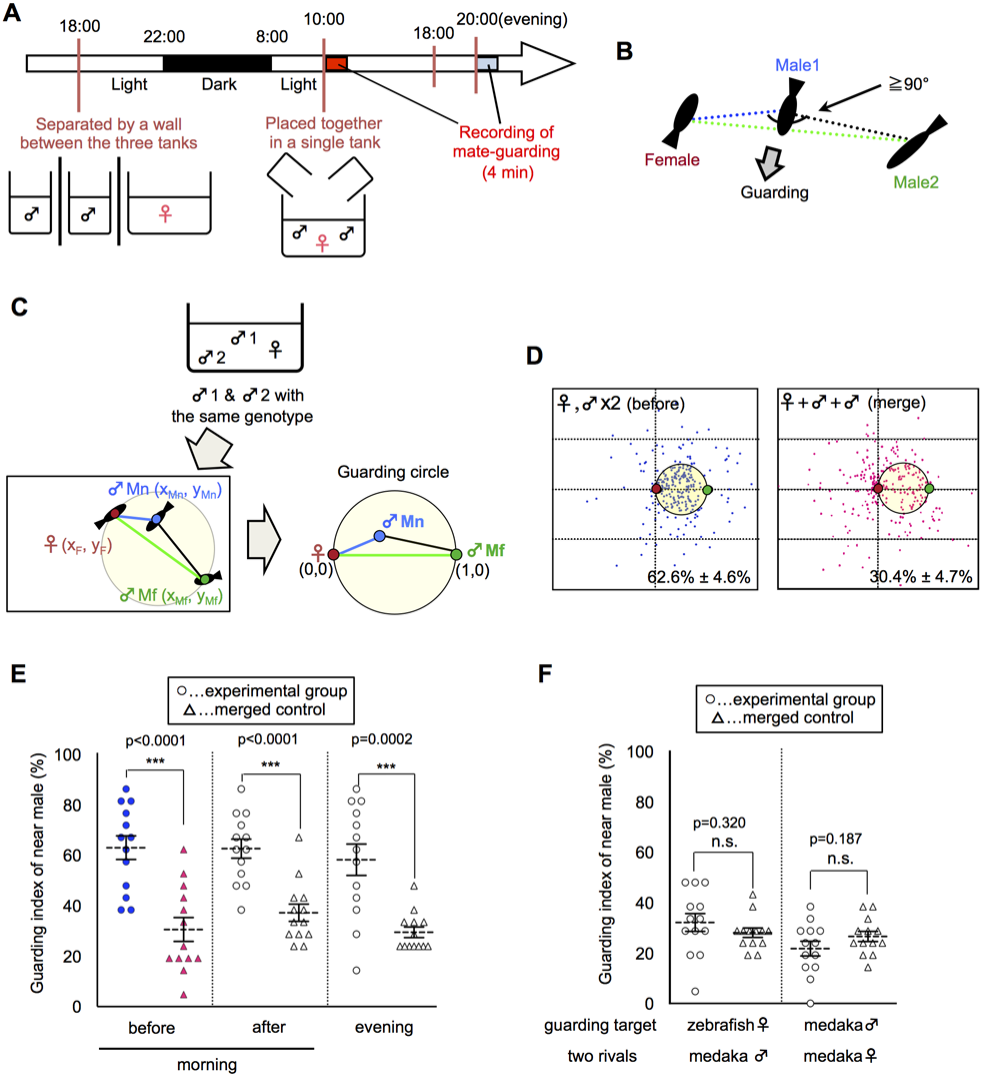
Emergence of mate-guarding in a triadic relationship. (A) Time-course for the mate-guarding behavior observation. One female and two males were placed in an aquarium in the morning (~10:00) or in the evening (~20:00). Lights were turned off at 22:00. (B) One example of typical mate-guarding behavior. One male tended to maintain its position between the female and the other male and prevent that male from approaching the female. We defined this behavior as “mate-guarding behavior”. (C) A procedure for calculating the guarding index representing the degree of mate-guarding. As fish with the same genotype were used, we discriminated the two male fish according to the mean distance for 100 s from the female (near male and far male). We measured the relative locations of the three fish and calculated the probability of the near male being in the guarding circle with center (1/2, 0) and radius 1/2 when the female and far male positions were defined as (0, 0) and (1, 0), respectively. We defined this probability as the “guarding index of the near male” and compared the groups in the guarding test. (D) Relative positions of the three fish (one female and two males). Dots indicate the relative positions of the near male over 100 s (1 dot/5 s). Red and green small circles indicate positions of the female and far male, respectively. Guarding indices are shown in the lower right. “Merge” indicates superimposed data of three fish moving independently, and thus were used as a negative control. Each n = 21 dots x 13 trials. (E) Comparison of guarding indices of the near male in the experimental group with those in the merged control to examine the effect of female condition on the guarding test. Mean ± SEM. Each n = 13, Student’s t-test: ***P<0.001. Before: guarding test before spawning in the morning. After: guarding test after spawning in the morning. Evening: guarding test in the evening. (F) Comparison of guarding indices of the near male in the experimental group with those of the merged control to examine the effect of fish properties on guarding test. Mean ± SEM. Each n = 13, Student’s t-test. Zebrafish: one zebrafish female and two medaka males. Female: two females and one male. Please see S3 Fig. for detail procedures for individual conditions.

We quantitatively analyzed the mate-guarding behavior by calculating the relative position of three fish in a behavioral assay (S1 Fig.). When two males and one female were allowed to swim together in the morning (Fig. 1A), the male-male competition led to the tendency of one male to maintain its position near the female and prevent the other male from approaching the female (S1–S2 Movies). We defined this behavior of the nearest male as “mate-guarding behavior” (Fig. 1B). To quantify the degree of mate-guarding, we generated a novel index to represent the degree of mate-guarding of the focal fish. First, we measured time-series coordinate data of the three individual medaka fish (2 males and 1 female) for 100 s and calculated the relative locations of the three fish. The relative positions of the focal male were calculated when the fixed positions of the female and the other rival male were defined as (0, 0) and (1, 0), respectively (Fig. 1C). We spotted the relative positions of the focal male during the 100 s (once every 5 s; S1 Fig.). We then calculated the probability of the focal male being within the “guarding circle”, defined as a circle with center (1/2, 0) and radius 1/2 (Fig. 1C). The presence of the focal male in the guarding circle indicated that the focal male occupied a dominant position, allowing him to both remain near the female and interfere with the rival (the other male). Thus, the probability of being within the guarding circle was considered to represent the degree of mate-guarding of the focal fish. Hereafter, we defined this probability as the “guarding index”.

To evaluate whether mate-guarding emerges in a triadic relationship, we examined whether the “guarding index” of the nearest of the two males significantly increases based on the interactions of the three fish (guarding test). The “near male” was defined as the male whose mean distance from the female during the 100-s recording period was shorter than that of the other male. The “far male” was defined as the other male. We then generated a “merged group” as a negative control, in which the three fish freely swam in individual aquaria without any social interaction. We separated three fish into three tanks of the same size, recorded the independent movement of each fish, and superimposed the data, which were used to calculate the virtual guarding index of the near male as a negative control. The guarding test using a wild-type medaka strain (drR) revealed a guarding index of 62.6% ± 4.6% for the near male, which was significantly higher than that of the merged control (30.4% ± 4.7%; Fig. 1D: before, merge). There were also significant difference of the guarding index between experimental and merged using different size (small, large) and shape (rectangular vs round) tanks (S2 Fig.), suggesting that mate-guarding robustly emerges irrespective of the geometric constraints of the apparatus. In most cases, medaka males remained near the female without performing an apparent quick-circle (i.e., the male’s courtship display) and interrupted the rival male without expressing aggressive behavior such as attack or bite [17, 18]. Thus, a unique behavioral repertoire in the male-male competition emerges in the triadic relationships.

### Behavioral properties of mate-guarding behavior in medaka fish

Mate-guarding in most species is considered to be a male-specific behavior in conspecific social groups [2–8]. Extended periods of mate-guarding (pre- or post-copulation) differ among species [2–8]. Here we investigated whether medaka fish exhibit the behavioral properties of mate-guarding. First, we examined whether medaka fish exhibit pre- or post-spawning mate-guarding. Sexually mature females have a 24-h reproductive cycle and spawn eggs once each morning [13–16]. We compared the guarding indices of the near male (guarding test) just before and after spawning in the morning (S3A and S3B Fig.). In addition, we performed the same test in the evening (S3E Fig.) to examine whether males exhibit mate-guarding in a time of day-dependent manner. All three indices (62.6% ± 4.6%, 62.3% ± 3.7%, and 57.9% ± 6.1%, respectively) were significantly higher than that for the negative control (merged data: 30.4% ± 4.7%, 37.0% ± 3.4%, and 29.3% ± 2.1%, respectively) (Fig. 1E), indicating that mate-guarding occurred irrespective of spawning.

We then examined whether mate-guarding emerged in the presence of females of other fish species. When the medaka female was replaced with a female zebrafish (S3C Fig.), the guarding index of the near male (31.9% ± 3.5%) was almost same as that of the merged control (27.8% ± 1.9%), suggesting that mate-guarding behavior is mediated by conspecific social cognition (Fig. 1F). Next we examined whether two medaka females exhibit mate-guarding toward a male (S3D Fig.). When two females and one male were placed in a single tank, the guarding index of the near female between the two females (21.6% ± 2.9%) was almost same as that of the merged control (26.4% ± 2.0%) (Fig. 1F). Furthermore, medaka males did not exhibit mate-guarding toward a male, and medaka females did not exhibit mate-guarding toward a female (S4 Fig.). In addition, medaka males did not exhibit significant mate-guarding toward sexually-immature females (S4 Fig.). Taken together, these results suggested that mate-guarding in medaka is a male-specific behavior toward sexually mature females.

Finally, we examined whether visual information is required for mate-guarding behavior. In medaka fish, social recognition in mating behaviors is mainly mediated by visual information [13, 15]. There was no significant difference between the guarding index of near males with a single eye removed and that of merged control (S5A Fig.). Single eye-removed males exhibit normal courtship behaviors [15], and thus the eye-ablation surgeries are assumed to not affect overall activity. In addition, medaka males exhibit significant mate-guarding toward females kept within a transparent cylinder tank that allows the males to only see the female without water intercirculating between the female’s enclosure and the male’s enclosure (S5B and S5C Fig.). Taken together, these findings suggest that visual sensory information is necessary and sufficient to elicit male mate-guarding, although the possibility that other sensory information (pheromones, touch) modulates this behavior as well could not be excluded.

### Positive correlation between dominance in mate-guarding and reproductive success

**Fig 2:**
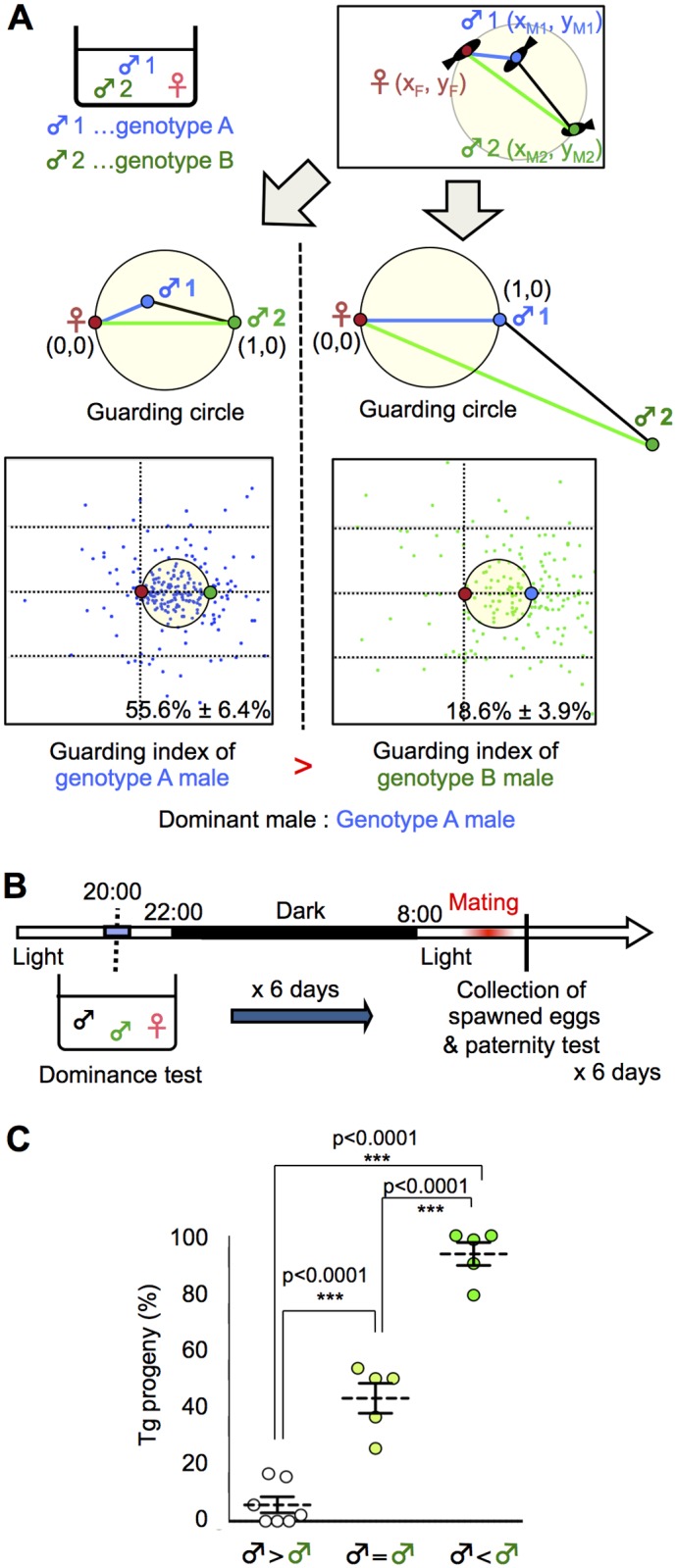
Dominance of mate-guarding in a triadic relationship. (A) Comparison of the dominance in mate-guarding behavior (dominance test). We used one *genotype A* male and one *genotype B* male and measured their mate-guarding behavior when exposed to a female. We measured the relative locations of the three fish and calculated the probability of the *genotype A* male being in the guarding circle when the female and *genotype B* male positions were defined as (0, 0) and (1, 0), respectively (Left). We defined this probability as the “guarding index of *genotype A*”. In contrast, we also calculated the probability of the *genotype B* male being in the guarding circle when the female and *genotype A* male positions were defined as (0, 0) and (1, 0), respectively (Right). We defined this probability as the “guarding index of *genotype B*” and compared with that of *genotype A*. A higher guarding index indicates higher dominance in the mate-guarding behavior compared with the other male. (B) Time-course for the paternity test. (C) Higher mating success rate of the dominant male. ♂ < ♂: Guarding index of Tg male significantly higher than that of the wild-type male prior to mating and vice versa, ♂ = ♂: No significant difference between the two males. Mean ± SEM. n's = 7, 5, 5, respectively. one-way ANOVA: Scheffé’s post-hoc ***P<0.001.

In various species, mate-guarding increases male reproductive success [6, 30, 31]. We examined whether dominance in mate-guarding positively correlates with male reproductive success. In the present study, we designed a “dominance test” to determine which male was dominant based on the guarding index. Fig. 2A shows a schema of the procedure used to determine the dominance of two males with different genotypes (A and B) in a triadic relationship. First, we measured the guarding index of a male with genotype A by calculating its relative position as described above. Then we measured the guarding index of a male with genotype B as the focal fish, and compared the “guarding indices” of two males with genotypes A and B (Figs. 2A and S6).

To examine whether a dominant male had high reproductive success, we performed a paternity test using two males with different genotypes (one wild-type and the other a transgenic [Tg] expressing green fluorescent protein [GFP] in the primordial germ cells [*homozygote olvas*:*gfp*]). GFP detection in the medaka embryo allowed us to genotype the progeny. The day before mating, we performed a dominance test using wild-type and Tg males, and the next morning we performed a paternity test on the fertilized eggs from the females (Fig. 2B). Medaka females have a single brood of 5 to 20 eggs each morning and, in most cases, the eggs are fertilized by the first male that exhibits ejaculation. Thus, we could determine which male won the competition for mating based on the paternity test [15]. We performed a dominance test using 17 groups for 6 days (1 test/day; S7 Fig.). When Tg males were judged to be dominant (5/17 groups), the percentage of Tg progeny was approximately 93.6%. When wild-type males were judged to be dominant (7/17 groups), the percentage of Tg progeny was approximately 5.7%. In the remaining 5 of the 17 groups, we could not determine dominance because there was no significant difference in guarding index between the two males (Fig. 2C). The combined results of the dominance test and paternity test revealed that the dominant male had a significantly higher reproductive success rate than the subordinate male.

### Essential role of V1a2 receptors for the emergence of mate-guarding

**Fig 3:**
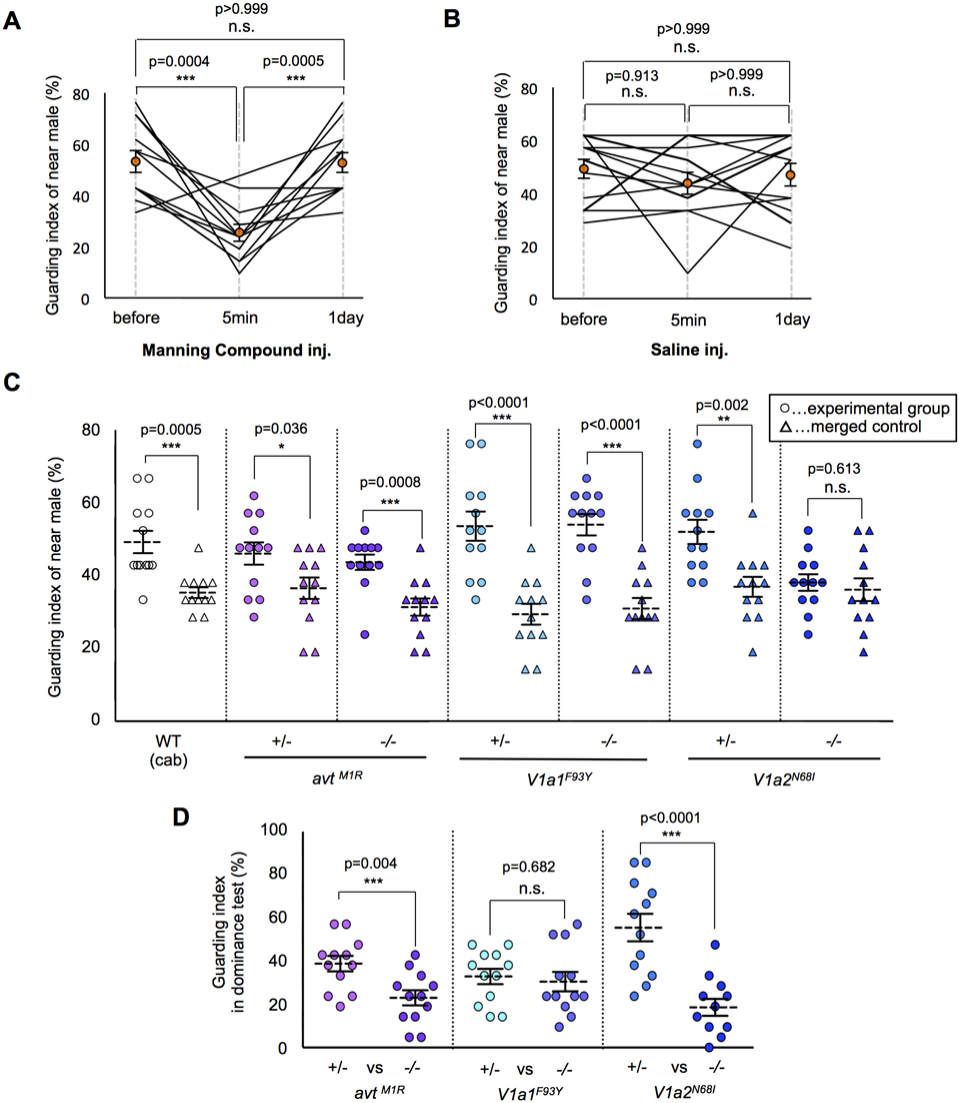
Effect of the AVT system on mate-guarding behavior. (A) Manning compound injection decreased the guarding index. Mean ± SEM. Each n = 12, one-way repeated measures ANOVA: Bonferroni correction, ***P<0.001. (B) Saline injection didn’t change the guarding index. Mean ± SEM. Each n = 12, one-way repeated measures ANOVA. (C) Effect of the AVT system on emergence of mate-guarding behavior (guarding test). *avt* (*avt* ^*M1R/M1R*^) and *V1a1* (*V1a1* ^*F93Y/F93Y*^) mutants exhibited mate-guarding behavior, whereas *V1a2* (*V1a2* ^*N68I/N68I*^) mutants did not. Mean ± SEM. Each n = 12, Student’s t-test: *P<0.05, **P<0.01, ***P<0.001. (D) Effect of the AVT system on dominance of mate-guarding behavior (dominance test). *avt* homozygote mutants (*avt* ^*M1R /M1R*^) and *V1a2* homozygote mutants (*V1a2* ^*N68I/N68I*^) tended to be subordinate males in the dominance test. Mean ± SEM. Each n = 12, Student’s t-test: ***P<0.001.

In prairie voles, the AVP system is suggested to be involved in mate-guarding, because AVP system mediates mate-induced selective aggression in a dyadic situation [20, 21]. **Here**, we found that injection of an AVT antagonist (Manning compound) impaired medaka mate-guarding in the triadic condition. The Manning compound is a commonly used antagonist of V1a receptors, including both subtypes V1a1 and V1a2 [32, 33]. We performed the guarding test using two males and one female, and then intraperitoneally injected an AVT antagonist or saline into the near male. At 5 min after the injection, we performed a second test using the same trio of fish. The guarding index of the injected fish in the second test was significantly lower than that in the first test (Fig. 3A-B and S3–S4 Movies). The AVT antagonist did not affect the overall activity of the injected males (S8A Fig. and S3–S4 Movies). In addition, the guarding index of the uninjected males significantly increased 5 min in the second test (S8B Fig.), while there was no effect of saline injection (S8C Fig.). This effect disappeared within 1 day after AVT antagonist administration. These findings suggested that AVT positively affected mate-guarding. We then examined the possible involvement of individual molecular components in the AVT pathway in mate-guarding by generating medaka mutants for genes encoding AVT [34, 35] and its receptors (V1a1 and V1a2) [36]. In fish species, two V1a-type receptors (V1a1 and V1a2) are expressed mainly in the brain [36–39], while V2-type receptors are prominently expressed in tissues other than the brain, such as gills, heart, and kidney [40]. Using the TILLING method, we identified *avt* mutants (*avt* ^*M1R/M1R*^) in which the first methionine residue was changed to arginine (S9A Fig.), *V1a1* mutants (*V1a1* ^*F93Y/F93Y*^) in which a conserved phenylalanine residue was changed to tyrosine (S11 Fig.), and *V1a2* mutants (*V1a2* ^*N68I/N68I*^) in which a conserved asparagine residue was changed to isoleucine (S12 Fig.). We confirmed that the 5’ untranslated region of the annotated *avt* transcripts was identical with that determined by 5’Race method (S9B Fig.). In addition, we performed mass spectrometry of AVT peptide based on matrix-assisted laser desorption/ionization–time of flight mass spectrometry (MALDI-TOF MS) (S10A and S10B Fig.) and selected reaction monitoring (SRM) (S10C and S10D Fig.), and demonstrated that there was no detectable AVT peptide in the brains of the *avt* mutants. To examine whether mate-guarding emerges between two mutant males with the same genotype, we performed the guarding test. The guarding test using the wild-type males (Cab strain) that were used for generating the mutants indicated a guarding index of 49.2% ± 3.1% for the near male, which was significantly higher than that of the merged control (35.3% ± 1.5% merge). The guarding index of the wild-type males was relatively lower than that in previous experiments (Fig. 1E), implying that external factors such as seasonal changes may affect our measurement of the guarding index of wild-type males. Interestingly, the guarding indices of the near male of the *avt* ^*M1R/M1R*^ and *V1a1* ^*F93Y/F93Y*^ mutants (43.7% ± 2.1% and 54.0% ± 3.0%, respectively) were significantly higher than those of the merged controls (31.3% ± 2.4% and 31.0% ± 2.9%, respectively), indicating that mate-guarding emerges between these mutant males (Fig. 3C). Thus, these two genes (*avt* and *V1a1*) were not required to elicit mate-guarding, suggesting that other ligands possibly activating the V1a2 receptor could compensate for the *avt* deficit. In contrast, there was no significant difference between the guarding indices of the near male among the *V1a2* ^*N68I/N68I*^ mutants (38.1% ± 2.3%) and that of the merged control (36.1% ± 3.1%) (Fig. 3C), indicating that mate-guarding did not occur between two *V1a2* mutant males (S5 Movie). The *V1a2* ^*N68I/N68I*^ mutation did not affect overall activity (S13 Fig.) and visual locomotion of the males (S14 Fig.). Thus, the V1a2 gene was required to elicit mate-guarding behavior. To confirm the behavioral phenotype of the loss-of-function mutations for the *V1a1* and *V1a2* genes, we generated *V1a1* and *V1a2* knockout mutant males using the TALEN method (S15 and S16A Figs.). The *V1a1* and *V1a2* knockouts have 4 and 7-bp deletions in the first exon, respectively. Both of the mutated transcripts encode C-terminal deleted proteins, lacking at least six of the seven transmembrane domains encoded in the first exon. Considering that the V1a1 and V1a2 receptors are seven-transmembrane receptors, the lack of six of the transmembrane domains should lead to a loss of function. The guarding index of the near male of the *V1a1* KO mutant (49.6% ± 2.7%) was significantly higher than that of the merged control (29.4% ± 4.5%). In contrast, there was no significant difference between the guarding index of the near male of the *V1a2* KO mutant (37.3% ± 3.8%) and that of the merged control (34.1% ± 1.9%) (S16B Fig.). Thus, these findings further supported that the loss of function of the V1a2 gene, but not the V1a1 gene, attenuated mate guarding.

### Essential role of the AVT ligand for dominance of mate-guarding

We then examined whether these mutations affected dominance of male-male competition in mate-guarding behavior. We performed dominance tests using two male siblings with a different genotype: one a homozygote and the other a heterozygote mutant (S6 Fig.). The guarding index of *avt* ^*M1R/M1R*^ (23.0% ± 3.5%) was significantly lower than that of heterozygote mutant (38.9% ± 3.6%), and that of *V1a2* ^*N68I/N68I*^ (18.6% ± 3.9%) was significantly lower than that of heterozygote mutant (55.6% ± 6.4%; Fig. 3D), indicating that *avt* ^*M1R/M1R*^ and *V1a2* ^*N68I/N68I*^ mutant males tended to be subordinate in male-male competition against their heterozygote mutant siblings. We also confirmed that the probability of greater proximity to the female of *avt* ^*M1R/M1R*^ and *V1a2* ^*N68I/N68I*^ males was significantly lower than that of heterozygote mutants (S17 Fig.). In contrast, the guarding index of *V1a1* ^*F93Y/F93Y*^ (34.9 ± 4.0%) did not differ significantly from that of *V1a1* ^*+/F93Y*^ (32.5 ± 4.1%; Fig. 3D), indicating that *V1a1* ^*F93Y/F93Y*^ mutant males show equivalent mate-guarding as the heterozygote mutants. Our results demonstrated that *avt* homozygote mutants exhibited decreased dominance against *avt* heterozygote mutants in mate-guarding, although male-male competition for mate-guarding occurred between two *avt* homozygote mutant males (Fig. 3C). Taken together, these findings suggest that AVT ligands enhance dominance of male-male competition in mate-guarding.

### Male sexual motivation toward the opposite sex and competitive motivation to the same sex in the mutants for AVT system

**Fig 4:**
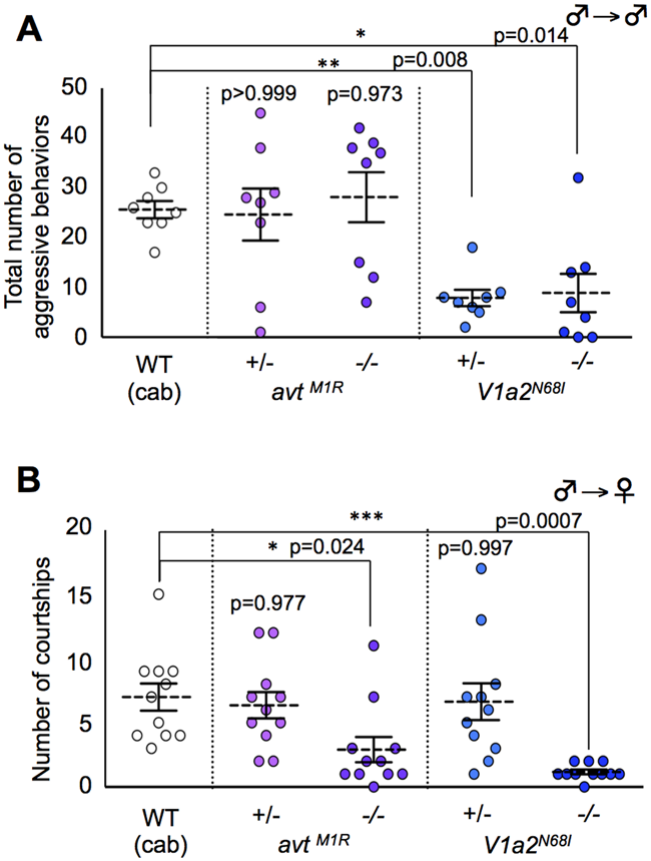
Effect of AVT-related genes mutations on aggressive behavior (intrasexual interaction) and courtship behavior (intersexual interaction). (A) *avt* mutant males exhibited normal aggression, whereas *V1a2* heterozygote and homozygote mutant males exhibited low aggression. Mean ± SEM. Each n = 8, Dunnett’s test: *P<0.05, **P<0.01 VS wild-type. (B) *avt* and *V1a2* mutant males showed lower motivation to mate than wild-type males. Mean ± SEM. Each n = 11, Dunnett’s test: *P<0.05, ***P<0.001 VS wild-type.

**Fig 5:**
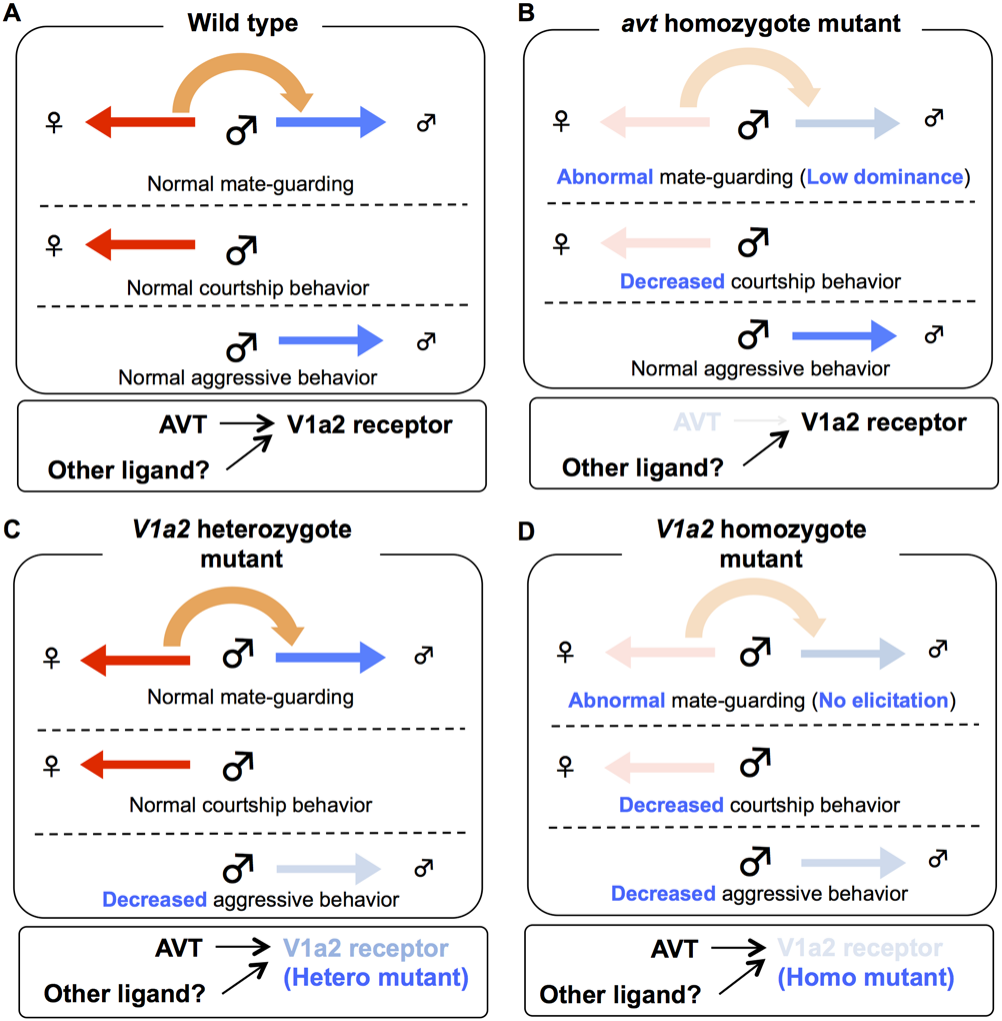
Possible model of AVT system in mate-guarding, aggressive and courtship behaviors. (A) Mate-guarding requires two different types of motivation: sexual motivation toward the opposite sex and competitive motivation toward the same sex, while courtship and aggressive behaviors require social motivation to either the opposite (red arrow) or same (blue arrow) sexes. When mate-guarding emerges in a triadic relationship, the presence of a potential mating partner may engage competitive motivation toward rival males via AVT system, leading to male-male competition (orange arrow). (B) The homozygote *avt* mutant males exhibited fewer courtship displays than wild-type males (Fig. 4B), whereas the mutant males exhibited normal aggression (Fig. 4A). Thus *avt* mutants normally have sexual motivation, but not competitive motivation toward the same sex. Decreased sexual motivation toward the opposite sex may cause low dominance of male-male competition in mate-guarding. *avt* was required just for dominance of mate-guarding. *avt* was not required for either normal aggressive behaviors or elicitation of mate-guarding, suggesting the presence of redundant system activating V1a2 receptor. (C) The heterozygote *V1a2* mutant males exhibited normal courtship displays (Fig. 4B), whereas they exhibited low aggression (Fig. 4A). Thus heterozygote *V1a2* mutants normally have competitive motivation, but not sexual motivation toward the opposite sex. Decreased competitive motivation towards the same sex has no effect on male-male competition in mate-guarding, because sexual motivation may dominantly drive the motivation for mate-guarding. The single functional *V1a2* allele may not produce enough of a gene product, leading to attenuated aggression. (D) The homozygote *V1a2* mutant males exhibited decreased courtship displays and low aggression (Figs. 4A and 4B). Thus homozygote *V1a2* mutants did not normally have social motivation to either the same sex or opposite sex. *V1a2* was required for elicitation of mate-guarding (Fig. 3C).

We examined whether these mutants have defects in sexual motivation toward the opposite sex and/or male competitive motivation toward the same sex that could cause mate-guarding abnormalities. To test this issue, we tested aggressive behavior elicited in groups comprising only males and male courtship behaviors in a male/female pair, respectively. The homozygote *avt* mutant males exhibited normal aggression in a non-mate guarding situation (Fig. 4A), whereas the mutant males exhibited fewer courtship displays than wild-type males (Fig. 4B), showing that *avt* mutants normally have competitive motivation to the same sex (rival males), but not to the opposite sex (a potential mating partner) (Fig. 5B). In contrast, the frequencies of aggressive behaviors and courtship display of homozygote *V1a2* mutant (*V1a2* ^*N68I /N68I*^) males were significantly lower than those of the wild-type control (Figs. 4A and B), indicating that the homozygote *V1a2* mutant did not normally have social motivation to either the same sex or opposite sex (Fig. 5D). Interestingly, the frequencies of aggressive behaviors of heterozygote *V1a2* mutant (*V1a2* ^*+/N68I*^) males were significantly lower than those of the wild-type control (Fig. 4A), while the heterozygote mutant males normally exhibited courtship displays (Fig. 4B), revealing that the heterozygote mutant males normally have sexual motivation, but not competitive motivation (Fig. 5C). The single functional *V1a2* allele might not produce enough of a gene product, leading to an attenuated aggression in a non-mate guarding situation. In conclusion, *avt* mutant males displayed defects in courtship display and did not normally exhibit mate-guarding (Fig. 5B). In contrast, mate-guarding behavior exhibited by heterozygote *V1a2* mutant males appeared normal despite defects in aggression (Fig. 5C). Based on these findings, the mate-guarding deficits of *avt* mutants were due to impaired sexual motivation, but not to impaired competitive motivation toward the same sex. In addition, behavioral analysis of *V1a1* mutant males suggested that *V1a1* is not required for either courtship or aggressive behavior (S18 Fig.), further implying a functional difference between *V1a1* and *V1a2* in social behaviors (S1 Table). In addition, AVT administration to wild-type males and *V1a2* heterozygote mutant increased the frequency of aggressive behaviors in a male group, but had no effect in *V1a2* homozygote mutant (S19 Fig.). Considering that *avt* mutants exhibit normal aggressive behaviors in a non-mate guarding situation, administration of exogenous AVT might artificially activate V1a2 receptors, which enhances male aggression.

## Discussion

Here we showed that mate presence drives male competitive motivation, leading to mate-guarding behaviors in a triadic relationship. The behavioral repertoire of the triadic relationship, however, differs between courtship behavior in a dyadic setup and aggressive behavior elicited in a male group. Mate presence in several species facilitates male aggressive behaviors [41–46]. For example, intrasexual competition in jacanas [45] and syngnathid fishes [46] requires mechanisms of selective attention to females. Apparent aggressive behaviors such as attacking and biting, however, were not enhanced in the triadic relationship of medaka fish. Thus, the unique behavioral repertoires (approaching behavior to a mating partner and interrupting behaviors to a rival male) emerge in the triadic relationships of medaka. Furthermore, the behavioral phenotypes in mate-guarding and aggressive behavior differed between the *avt* and *V1a2* mutants (Figs. 5B and D). *V1a2* was required for the emergence of mate-guarding among mutant males, whereas *avt* was not required (Fig. 3C). *avt* was required just for the dominance of mate-guarding (Fig. 3D). The double functional *V1a2* allele was required to elicit aggressive behavior to the same degree as the wild-type, while the *avt* mutation did not significantly affect this behavior (Fig. 4A). These findings implied that V1a2 receptors are better conserved and more central in these signaling systems than the ligands and that some compensatory systems activate the V1a2 receptors in *avt* mutants. AVT may not be the only ligand that activates the V1a2 receptors (Fig. 5). The involvement of AVT/AVP ligands in aggressive behaviors has been investigated based on pharmacologic manipulations in various vertebrates from fish to rodents [22, 23, 25, 47–49]. Administration of exogenous ligands (AVT) into the brain enhances aggression, while AVT/AVP receptor V1a antagonist suppresses aggression. In medaka, AVT administration also enhanced aggression (S19 Fig.). These findings based on pharmacological analysis, however, do not preclude the possibility that other endogenous ligands activate the V1a receptors. Our findings are consistent with a recent rodent study using a rat natural mutant with vasopressin (AVP) deficiency [49], which revealed that a loss of function of the AVP gene does not affect aggressiveness, especially in reproductively experienced males. Thus, the differences in behavioral phenotypes between *avt* and *V1a2* mutants may be due to the existence of redundant systems that activate the V1a2 receptors in medaka fish brain. Isotocin, a non-mammalian homolog of oxytocin, is a candidate endogenous ligand that activates V1a2 receptors. Isotocin has affinity for the teleost vasotocin receptor [50] and there is significant cross-talk between oxytocin, AVP and their receptors in mammals [51]. Further analysis of isotocin and its mutants is required to understand the signaling pathways that activate V1a2 receptors.

Our findings suggest that the AVT system is involved in the process in which mate presence drives sexual motivation towards the opposite sex, which facilitates male competitive motivation for mate-guarding in the triadic relationship (Fig. 5A). Our finding is consistent with previous studies in the bluehead wrasse (*Thalassoma bifasciatum*) and African cichlid (*Astatotilapia burtoni*) [25, 52]. Pharmacological manipulations of the AVT system in both of the two fish species facilitate male courtship and territorial aggression behaviors that are associated with social dominance [24, 39]. In non-fish species such as bird and frog, involvement of AVT system in courtship and territorial aggression also has been suggested [53–55]. In mammalian species, the socially monogamous prairie vole (*Microtus ochrogaster*) has been used as a model organism for investigating the neurobiology of this type of complex social behavior, such as pair-bonding, which involves both intersexual and intrasexual interactions [56–58]. Prairie voles exhibit pair-bonding behavior involving affiliation toward a mate and agonistic behavior toward non-mates. A series of studies using prairie voles revealed that AVP and the V1a receptor subtype have essential roles in pair bonding and other behaviors associated with monogamy [20, 21, 56–58]. In the monogamous vole, however, each of the behavioral components (affiliation toward mates and agonistic behavior toward non-mates) was analyzed individually in the dyadic setup [56–58], and possible involvement of the AVT/AVP system in the actual mate-guarding behavior in the triadic relationship has not yet been investigated in prairie voles. Establishing a quantitative assay system for this behavior under laboratory conditions allowed us to genetically study the molecular mechanisms underlying mate-guarding behavior in a triadic experimental setup, but not dissect the sub-behaviors, which have been studied for several decades.

Furthermore, it should be noted that the AVT receptor V1a2 has a central role in regulating fish social behaviors such as mate-guarding, courtship behaviors, and aggressive behaviors. The AVT receptor function in fish species has been investigated based on pharmacologic manipulations alone, and the selectivity of mammalian V1a receptor antagonists like the Manning compound for the two V1a-type receptors (V1a1 and V1a2), is unknown [22–25, 33, 50]. Thus, functional differences between the two receptors cannot be determined based on pharmacologic studies alone. Some recent studies reported differential gene expression between *V1a1* and *V1a2*. In the larval brain of zebrafish, the two genes are expressed in the same brain regions, but few neurons coexpress *V1a1* and *V1a2* [37]. The differential regulation of gene expression levels in the bluehead wrasse *Thalassoma bifasciatum* implies the importance of *V1a2* over *V1a1* in fish social behavior [36]. Our findings suggested differences in the behavioral function between *V1a1 and V1a2* in mate-guarding.

In adult fish brains, *V1a2*-expressing neurons are broadly located in various brain regions, such as the dorsal and ventral telencephalon, and the preoptic area [36, 37, 39], that are thought to be important for social decision-making [59]. It remains unknown, however, how *V1a2*-expressing neurons mediate individual social components in different social contexts. More importantly, it should be noted that the gene knockout method eliminates AVT or its receptors in the relevant social behavior-related neural areas, but also more widely in the brain and other tissues such as the gonads (which also express AVT receptors) throughout development. Further studies are needed that selectively manipulate subpopulations of *V1a2*-expressing neurons in the adult brain. Genetic mosaic techniques are available in medaka fish to visualize and/or genetically modify a neuronal subpopulation within complex neural circuits [60, 61]. Genetic dissection of the AVT system using such advanced molecular genetic methods will allow us to identify the microcircuits that regulate social behaviors. The present study using medaka mutants is an important first step toward unveiling this complex neuromodulatory pathway, for which our current understanding is very poor.

## Materials and Methods

### Ethics statement

The work in this paper was conducted using protocols approved by the Animal Care and Use Committee of the University of Tokyo (permit number: 12–07). All surgery was performed under anesthesia using MS-222, and all efforts were made to minimize suffering, following the NIH Guide for the Care and Use of Laboratory Animals.

### Fish and breeding conditions

Medaka fish (*Oryzias latipes*; drR strain, Cab strain, mutants, Tg(*homozygote olvas*:*gfp*), were maintained in groups in plastic aquariums (13 cm x 19 cm x 12 cm (height)). TILLING mutants were provided by the Medaka National BioResource Project (http://www.shigen.nig.ac.jp/medaka/). All fish were hatched and bred in our laboratory. Sexually mature male (2.5 cm~3.2 cm, 240 mg~330 mg) and female (2.7 cm~3.1 cm, 370 mg~400 mg) adult medaka 3~5 months of age producing fertilized eggs every morning were used at least once a week for the behavior assay. The water temperature was ~28°C and light was provided by standard fluorescent lamps for 14 h per day (08:00–22:00).

### Mate-guarding behavior assay

A detailed procedure is provided in S1 Fig. One female and two males were placed in an aquarium (water depth was about 3–4cm, the light intensity was about 400–500 lx), and their behavior was recorded from the bottom of the aquarium, in the morning (10:00 to 12:00) and in the evening (20:00 to 21:00). Light was provided for 14 h per day (08:00 to 22:00). As a negative control group (merged group), we performed the same experiment using virtually merged trios, recording one female and two males, each placed in a separate aquarium (“Merge”). We converted video files into 21 image sequences per 5 s, and manually spotted the head and tail positions of the three medaka fish using ImageJ (NIH, Bethesda, MD, USA) to calculate the center positions as the body positions. The male whose mean distance from the female was shorter than that of the other male was “the near male” and the other was “the far male”. Based on the positions of the female (x_F_, y_F_), the far male (x_Mf_, y_Mf_), and the near male (x_Mn_, y_Mn_), the relative positions of the near male (X, Y) were calculated by the following formula when the female and far male positions were defined as (0, 0) and (1, 0), respectively (See S1 Fig.).

$$X=\frac{\{\left(x_{Mf}-x_{F}\right)\left(x_{Mn}-x_{F}\right)+\left(y_{Mf}-y_{F}\right)\left(y_{Mn}-y_{F}\right)\}\sqrt{{{(x}_{Mn}-x_{F})}^{2}+{{(y}_{Mn}-y_{F})}^{2}}}{{\left\{{{(x}_{Mf}-x_{F})}^{2}+{{(y}_{Mf}-y_{F})}^{2}\right\}}^{\frac{3}{2}}}$$

$$Y=\frac{\{\left(x_{Mf}-x_{F}\right)\left(y_{Mn}-y_{F}\right)-\left(y_{Mf}-y_{F}\right)\left(x_{Mn}-x_{F}\right)\}\sqrt{{{(x}_{Mn}-x_{F})}^{2}+{{(y}_{Mn}-y_{F})}^{2}}}{{\left\{{{(x}_{Mf}-x_{F})}^{2}+{{(y}_{Mf}-y_{F})}^{2}\right\}}^{\frac{3}{2}}}$$

We spotted the relative positions of the near male and defined a circle with center (1/2, 0) and radius 1/2 as the “guarding circle”. When the near male was within the guarding circle, the angle between the vectors from the near male to the female and from the near male to the far male was obtuse. The probability of being in the guarding circle was defined as the “guarding index” (See S1 Fig.).

### Dominance test

A detailed procedure is provided in Fig. 2A. We used one *genotype A* male and one *genotype B* male and evaluated their mate-guarding behavior in the presence of a female. We measured the relative locations of the three fish and calculated the probability of the *genotype A* male being in the guarding circle when the female and *genotype B* male positions were defined as (0, 0) and (1, 0), respectively (Left). We defined this probability as the “guarding index of *genotype A*”. In contrast, we also calculated the probability of the *genotype B* male being in the guarding circle when the female and *genotype A* male positions were defined as (0, 0) and (1, 0), respectively (Right). We defined this probability as the “guarding index of *genotype B*” and compared with that of *genotype A*. A higher guarding index indicates higher dominance in the mate-guarding behavior compared with the other male (Fig. 2A).

### Paternity and dominance tests

The paternity test was performed as described previously [15]. A detailed procedure is provided in Figs. 2B and S7. One female and two males, one of which was the drR wild-type strain and the other of which was Tg (*homozygote olvas*:*gfp*), were used for this test. To determine which male was dominant, we performed a dominance test over 6 days using same three fish (6 trials). The male whose mean guarding index for the 6 trials was significantly higher was considered dominant and the other was considered subordinate (S7 Fig.). Tg(*olvas*:*gfp*) were distinguished from drR by the GFP fluorescence of primordial germ cells, visible at 3 days post-fertilization, and even in the ventral area of the adult, by fluorescent microscopy (MZ16F, Leica, Tokyo, Japan). Therefore, we genotyped the progeny based on GFP detection.

### Manning compound administration

This procedure was performed as previously described [25] with minor modifications. After the guarding test, we anesthetized the near males using MS-222 and intraperitoneally injected the Manning compound. We injected 3.2 μg Manning compound/g body weight.

### TILLING and high-resolution melting curve experiments

This procedure was performed as previously described [26]. To amplify the *avt*, *V1a1*, and *V1a2* locus that includes the final gene product, we performed polymerase chain reaction (PCR) with *avt-*specific primers: 5′- AGACGTCCACACCGACA-3′ and 5′- GCCAAAAGCATCTCACCT-3′, and *V1a1-*specific primers: 5′- GGACAGCCTTTGCAACTT-3′ and 5′- GTTTGTGGAGGAGAGGGTA-3′, and *V1a2-*specific primers: 5′- CAGCGTGCTGCTCTTGA-3′ and 5′- CGATGTAACGGTCCAAAGT-3′. The PCR conditions were as follows: 1 cycle of 94°C for 2 min, followed by 45 cycles of 94°C for 15 s; annealing at 63°C (*avt*) or 60°C (*V1a1*, *V1a2*) for 30 s, and then at 68°C for 30 s (*avt*, *V1a2*) or 60 s (*V1a1*); and a final denaturing and re-annealing step (1 cycle of 94°C for 30 s, followed by rapid cooling to 28°C). Each of the 5771 PCR products derived from genomic DNAs was subjected to the high-resolution melting assay. Based on differences in the melting curves, mutant candidates were selected. Melting curves were analyzed using the LightScanner (Idaho Technology, Salt Lake City, UT, USA), as previously described [26]. The mutations were then identified by sequencing the PCR product of the second positive genomic DNAs using BigDye Terminator version 3.1 (Applied Biosystems, Foster City, CA, USA) and the ABI 3730XL sequencing platform. We backcrossed the TILLING mutants with Cab fish three times and crossed those fish to generate the homozygote mutants.

### 5’-RACE of *avt*

Total RNA was extracted from the male medaka brain (drR and Cab strains) using TRIZOL Reagent. 5’-Rapid amplification of cDNA ends (5’-RACE) was performed using a SMARTer RACE cDNA Amplification Kit (Clontech) following the manufacturer’s instructions. The amplification was performed using the Universal Primer A mix and 5’- TGATCCCAGCCTCCGGCAAT-3’ (*avt* gene specific primer). The amplified PCR products were cloned into a pGEMT Easy Vector (Promega) and sequenced. We sequenced 10 cDNA clones derived from the drR strain and 9 cDNA clones derived from the Cab strain and confirmed that the sequences of all 19 cDNA clones started from the transcription initiation site, which was predicted based on the annotated *avt* sequence.

### Mass spectrometry (MALDI-TOF MS and SRM analysis)

Mass spectrometry (MS) was performed as described previously with minor modifications [62, 63]. Peptides in the pituitary of wild-type (Cab) and *av*t mutant strains were extracted using 0.1% (v/v) trifluoroacetic acid (TFA). After concentration and purification with a self-made C18 STAGE tip [62, 64], 5 μl of sample/one pituitary in 0.1% (v/v) TFA solution was analyzed using MS. To create peptide profiles of the pituitary of wild-type (Cab) and *av*t mutant strains, we performed MALDI-TOF MS analysis using an AXIMA TOF^2^ mass spectrometer (Shimadzu biotech, Kyoto, Japan) [34, 63]. The peptide solution (0.5 uL) was mixed with 0.5 uL matrix solution [2% (w/v) α-cyano-4-hydroxycinnamic acid in 50% (v/v) acetonitrile/0.1% (v/v) TFA] and spotted on a stainless-steel MS sample plate. To confirm the expression level of the avs peptide, SRM analysis was performed on a QTRAP5500 mass spectrometer coupled to an Eksigent nanoLC-Ultra system via a cHiPLC-nanoflex module (AB SCIEX, Framingham, MA, USA). Peptides were separated on a nano cHiPLC C18-reversed phase column (Chrome XP C18CL, 75 μm ID × 15 cm) and eluted at a constant flow rate of 300 nL/min. A linear gradient (2%−50% mobile phase B) was applied for 15 min, followed by a 6-min wash with 90% mobile phase B; the column was then equilibrated for 20 min with 2% mobile phase B.

### TALEN experiment

TALEN experiments were performed as described previously [28, 29]. Potential TALEN target sites in the locus were searched using the TALEN Targeter program (https://tale-nt.cac.cornell.edu/node/add/talen). TAL repeats were assembled using the Golden Gate assembly method with slight modifications. Expression vectors for the TALENs were linearized by digestion with *NotI*. Capped RNAs were synthesized using the mMessage mMachine SP6 Kit (Life Technologies, Gaithersburg, MD, USA) and purified using the RNeasy Mini Kit (Qiagen, Valencia, CA, USA). Pairs of RNA for the TALENs (150 ng/μl) were injected into fertilized eggs of the drR strain by a microinjection method.

### Prediction of secondary structure of V1a receptors

We obtained the genomic sequence information of V1a receptors from the Ensemble medaka genome browser (http://www.ensembl.org/Oryzias_latipes/Info/Index) and predicted their secondary structure using “SOSUI”, which is a program that predicts transmembrane regions from amino acid sequences (http://bp.nuap.nagoya-u.ac.jp/sosui/).

### Visual response and locomotion ability test (Optomotor response)

We assessed the optomotor response of the *avt* ^*M1R*/*M1R*^, *V1a2* ^*+/N68I*^, and *V1a2* ^*N68I/N68I*^ fish using our previously described apparatus [65] (see S14 Fig.). The medaka were placed in a fixed 15-cm-diameter circular tank, which was placed within a striped 20-cm-diameter cylinder. The depth of water in the tank was 2 cm. The striped cylinder was positioned on a rotatable metal disk that was driven by a motor, IHT6P3 (SERVO, Kiryu, Japan), that could move in either direction and at various speeds using the C-30PN (SERVO) motor driver. We recorded the optomotor response of medaka using a CCD camera (XC-ST70; SONY, Tokyo, Japan) and extracted the position of the medaka and stripes. A series of frames was analyzed using the software Move-tr/2D 7.0 (Library, Tokyo, Japan).

### Courtship behavior assay

This procedure was performed as previously described [15]. Males and females were separated in the evening (18:00–19:00) the day before the assay. The mating pair was then placed together in a single tank (the light intensity was about 600–700 lx under breeding condition) the next morning, and mating behavior was recorded for 5 min. We counted the number of courtship displays. We performed a quality check on female reproductive states following our previously described procedure [15]. We determined whether the females had spawned fertilized eggs 30 min after recording the movie. If the females had not produced fertilized eggs at that time, we judged that the females were not in a reproductive state, which might be due to stress or lack of food. The percentage of females in a reproductive state was 65%~75% and we did not analyze the data of the females not in the reproductive state.

### Aggressive behavior assay

This behavioral assay was performed as previously described [17, 18] with minor modifications. We placed three males of the same strain into a single tank (13 cm x 19 cm x 12 cm (height)). Water depth was about 7–8cm, the light intensity was about 600–700 lx under breeding condition and we allowed them to adapt to the apparatus for 60~70min. Movement of each fish was recorded for 5 min. We defined three behavioral components, “bite” and “attack” [17, 18] as “aggressive behaviors”. The difference between “aggressive behaviors” in the previous work [17, 18] was that in the present study we did not consider approaching and threatening behaviors that did not include touching each other (chase, replace, and frontal–lateral display) as “aggressive behaviors”, because it is very difficult to discriminate these behaviors from shoaling-like behavior that *V1a2* mutants frequently exhibit in the male group. We counted aggressive behaviors of three fish (Figs. 4A, S18) or the focal fish (S19 Fig.).

## Supporting Information

S1 Fig Procedure for the mate-guarding behavior assay. Upper left: Set-up for recording mate-guarding behavior. Fish movement was recorded from underneath, as the tank is transparent. Upper right: Time-course for the behavioral test. We separated the three fish before the behavioral test, and then put the three fish into the same tank for the behavioral measures to control the timing of spawning. We determined the time (~10:00) for the behavioral test following an established method for mating assay published in our previous study [15]. Lights were turned off at 22:00. We converted video files into 21 image sequences per 5 s, and manually measured the head and tail positions of the three medaka fish using ImageJ (NIH) to calculate the center positions, which were used as their body positions. The male with a shorter mean distance for 100 s from the female than the other male was defined as the “near male”, and the other male was defined as the “far male”. Based on the positions of the female (x_F_, y_F_), the far male (x_Mf_, y_Mf_), and the near male (x_Mn_, y_Mn_), the relative positions of the near male (X, Y) were calculated by the formula described in the text when the positions of the female and the far male were defined as (0, 0) and (1, 0), respectively. We spotted the relative positions of the near male and defined the “guarding circle” as a circle with center (1/2, 0) and radius 1/2. When the near male is present in the guarding circle, the near male remains near the female and interferes with the rival (the far male). Thus, we defined the probability of being in the guarding circle as an index representing the degree of mate-guarding (guarding index).
(TIFF)

S2 Fig Guarding tests using different tanks. The size (small, 9.5 cm x 13 cm x 12 cm (height); large, 21 cm x 30 cm x 10 cm (height)) and shape (15-cm diameter circular tank) of the tank did not influence this behavior. Water depth was about 3–4 cm. Mean ± SEM. Each n = 12, Student’s t-test: *P<0.05, **P<0.01, ***P<0.001.
(TIFF)

S3 Fig Time schedule of guarding tests. (A) “Before”: two males and a female were separated in the evening on the day before the assay and the next morning were placed together in a single tank. (B) “After”: two males and a female were separated in the evening on the day before the assay. The next morning, we allowed a third male, which was not used in the guarding test to mate with the female. After that, we removed the male for mating and placed two males (separated on the day before the assay) and one female together in a single tank. (C) “Zebrafish”: two medaka males and a zebrafish female were separated in the evening on the day before the assay and the next morning, were placed together in a single tank. (D) “Female”: two females and a male were separated in the evening on the day before the assay and the next morning were placed together in a single tank. (E) “Evening”: two males and a female were separated in the evening and after ~2 hours were placed together in a single tank (20:00–21:00).
(TIFF)

S4 Fig Guarding tests in various combinations of a guarding target and two rivals. We used a male instead of a female as a guarding target (left), two females instead of two males as rivals (middle), and an immature female (about 2 months old, 2.1 cm-2.2 cm, 130 mg–140 mg) instead of a mature female (right) as a guarding target. In all the cases, two rivals did not exhibit mate-guarding toward a guarding target. Mean ± SEM. Each n = 12, Student’s t-test.
(TIFF)

S5 Fig Requirement and sufficiency of visual information in mate-guarding behavior. (A) Unilateral and bilateral eye-ablated males did not exhibit mate-guarding. Sham fish were injured just above the eyes. Single and Double: One or two eyes were removed, respectively. Mean ± SEM. Each n = 12, Student’s t-test: ***P<0.001. (B) Procedure for the male or female isolated mate-guarding experiments, we placed a small transparent tank (6 cm diameter circular tank) in the center of the test tank. A female or male was placed in the small circular tank and two other males were placed in outside of the small tank (the water in the outer tank did not contain pheromones of the fish in the small tank). (C) Visual information is sufficient for males to exhibit mate-guarding. Mean ± SEM. Each n = 12, Student’s t-test: **P<0.01.
(TIFF)

S6 Fig Difference between the guarding test and dominance test. In the guarding test, we used two males with the same genotype, so we could judge whether or not the males exhibited mate-guarding behavior. In this test, however, the mate-guarding can emerge irrespective of the strength of used males, as the guarding indices are altered according the strength of the rival males. In the dominance test, we can directly compare the guarding indices between two different genotypes, because a triadic setup comprises two males with different genotypes. For example, in the guarding test using *avt* mutants (Fig. 3C), the guarding index of near males of the two weak males in the dominance test (*avt* homozygote mutants) was as high as that of the two strong males in the dominance test (*avt* heterozygote mutants), because the rival males were also weak in a triadic setup comprising the two weak males. Thus we cannot directly compare the guarding indices across different genotype based on guarding test.
(TIFF)

S7 Fig Procedure for the dominance and paternity tests. (A) We integrated the results of a 6-d dominance test and compared the guarding index of each genotype and judged which male was dominant by Mann-Whitney U test. (B) Pair-1 is shown as an example. The guarding index of the wild-type (drR) male (43.7%) was significantly higher than that of the transgenic (Tg; *homozygote olvas*:*gfp*) male (11.1%; Mann-Whitney U test, *P* = 0.020, n = 6). In this case, we judged that the wild-type (drR) male was dominant. If there was no significant difference (Mann-Whitney U test, *P*>0.05, n = 6), we judged that the two fish were equal. In Fig. 2C, 17 pairs were classified into three groups: the wild-type dominant pairs (n = 7, ♂ > ♂), Tg(*homozygote olvas*:*gfp*)-dominant pairs (n = 5, ♂ < ♂), and equivalent pairs (n = 5, ♂ = ♂). In Fig. 2C, we compared the percentage of GFP-positive eggs, indicating the Tg progeny rate, among the three groups.
(TIFF)

S8 Fig Effect of the Manning compound injection on general activity and dominance in mate-gurading. (A) The velocities of near males were not changed by injecting the Manning compound. Mean ± SEM. Each n = 12, Paired t-test. (B) The guarding indices of the far males (uninjected males) were significantly increased by injecting the Manning compound into the near males (5 min after injection) and this tendency disappeared 1 day after the injection. Mean ± SEM. Each n = 12, one-way repeated measures ANOVA with Bonferroni’s correction for multiple comparisons. **P<0.01 (C) The guarding indices of far males (uninjected males) were not altered by saline injection into near males. Mean ± SEM. Each n = 12, one-way repeated measures ANOVA with Bonferroni’s correction for multiple comparisons.
(TIFF)

S9 Fig Screening for *avt* mutant alleles by TILLING. (A) A local sequence dataset comparing the wild-type *avt* (*avt* ^+/+^) and avt^M1R^ homozygotes (*avt* ^*M1R*/*M1R*^) demonstrating the *avt* T2G mutation in avt^M1R^ mutants (black arrowhead). (B) A local sequence of 5’-RACE product confirming the transcription initiation site of *avt*, which was predicted by the annotated *avt* sequence. We sequenced 10 and 9 cDNA clones derived from the drR and cab strains, respectively and confirmed that the sequences of all 19 cDNA clones started from the transcription initiation site, which was predicted by the annotated *avt* sequence. “adaptor”: added nucleotide in SMARTer RACE cDNA Amplification Kit (Clontech).
(TIFF)

S10 Fig Mass spectrometry analysis (MALDI-TOF MS analysis and SRM assay). AVT peptides are present in the pituitary of the wild-type (Cab), but not in the *avt* mutant. MALDI-TOF MS spectra of the peptides from the pituitary in the wild-type (A) and *avt* mutant (B) brains. The x-axis shows the m/z, mass to charge ratio; the y-axis shows the intensity of the molecular ions. An ion peak at m/z 1050.5 indicated the presence of the AVT peptide in the wild-type (A). For the SRM assay, we selected Q1 (precursor ion: 525.8)/Q3 (fragment ion y3: 328.2), based on tandem MS spectrum of [Arg8]-vasotocin (Sigma Aldrich, V0130). Abundant AVT peptides were detected in the wild-type (C), while no AVT peptide was detected in *avt* mutant (D). The x-axis shows retention time; the y-axis shows the intensity of the molecular ions.
(TIFF)

S11 Fig Screening for *V1a1* mutant alleles by TILLING. A local sequence dataset comparing the wild-type *V1a1* (*V1a1* ^+/+^) and V1a1^F93Y^ homozygotes (*V1a1* ^*F93Y/F93Y*^) demonstrating the *V1a1* T278A mutation in V1a1^F93Y^ mutants (black arrowhead).
(TIFF)

S12 Fig Screening for *V1a2* mutant alleles by TILLING and the primary structure of V1a receptor paralogs. (A) A local sequence dataset comparing the wild-type *V1a2* (*V1a2* ^+/+^) and V1a2^N68I^ homozygotes (*V1a2* ^*N68I/N68I*^) demonstrating the *V1a2* A203T mutation in V1a2^N68I^ mutants (black arrowhead). (B) The primary structure of V1a receptor paralogs in mouse and fish. Phenylalanine 93 and arginine 68, which are identical among known forms, were changed to tyrosine and isoleucine in V1a1^F93Y^ and V1a2^N68I^ mutant alleles, respectively (red letters).
(TIFF)

S13 Fig Normal free swimming velocity in *avt* ^*M1R*/*M1R*^, *V1a2* ^*+/N68I*^, and *V1a2* ^*N68I/N68I*^ male fish. One minute after we placed 1 fish in the tank, we calculated its movement velocity for 60 s. Mean ± SEM. Each n = 4, Dunnett’s test.
(TIFF)

S14 Fig Normal optomotor response (OMR) in *avt* ^*M1R*/*M1R*^, *V1a2* ^*+/N68I*^ and *V1a2* ^*N68I/N68I*^ male fish. (A) Equipment for the analysis of the OMR described previously [65]. (B-E) Representative examples of traces of fish movement during OMR. (F-I) Integrated angular velocity during 60 s of (F) wild-type (Cab), (G) *avt* ^*M1R*/*M1R*^, (H) *V1a2* ^*+/N68I*^, and (I) *V1a2* ^*N68I/N68I*^ fish. Each line indicates integrated angular velocity of five individual fish. (J) Ratio of the mean fish angular speed to that of the stripe speed. Mean ± SEM. Each n = 5, Dunnett’s test.
(TIFF)

S15 Fig Generation of *V1a1* mutant alleles by TALEN. A local sequence dataset comparing wild-type *V1a1* (*V1a1* ^+/+^) and V1a1 knockout (KO) homozygotes demonstrating that a 4-bp deletion generated a nonsense mutation (G26X) in *V1a1* KO mutants. *V1a1* gene consists of two exons. The deletion was located in the first exon and the mutated transcripts encode C-terminal deleted proteins lacked six of the seven transmembrane domains encoded by the first exon.
(TIFF)

S16 Fig Generation of *V1a2* mutant alleles by TALEN and mate-guarding behavior of AVT receptor knockout mutants. (A) A local sequence dataset comparing the wild-type *V1a2* (*V1a2* ^+/+^) and *V1a2* knockout (KO) homozygotes demonstrating that a 7-bp deletion generated a nonsense mutation (D24X) in *V1a2* KO mutants. *V1a2* gene consists of two exons. The deletion was located in the first exon and the mutated transcripts encode C-terminal deleted proteins lacked six of the seven transmembrane domains encoded by the first exon. (B) *V1a1* KO mutants exhibited mate-guarding behavior, whereas *V1a2* KO mutants did not. Mean ± SEM. Each n = 12, Student’s t-test: **P<0.01, ***P<0.001.
(TIFF)

S17 Fig Dominant males in the mate-guarding behavior maintained closer proximity to the female than subordinate males. We judged which male was closer to the female in a total 256 images (21 images x 12 trials) in the dominance test (Fig. 3D). Each trial contained 21 images (5-s interval for 100 s. See S1 Fig.). Here we calculated the probabilities of being closer to the female between heterozygote and homozygote mutants based on the 256 images. We then detected a significant bias between the two probabilities using the chi-square test in *avt* (A) and *V1a2* mutants (C), but not in *V1a1* mutants (B).
(TIFF)

S18 Fig Lack of effect of *V1a1* mutation on aggressive behavior or courtship behavior. *V1a1* ^*F93Y*^ homozygote mutant males exhibited normal aggressive behavior (A) and courtship behavior (B). Mean ± SEM. Each n = 8 (A), n = 11 (B), Mann-Whitney U-test.
(TIFF)

S19 Fig Effect of AVT administration on aggressive behavior. AVT or saline was injected to the one of three fish intraperitoneally and the number of aggressive behaviors of focal male toward other males before and after injection was counted. (A, C, E) Injection of AVT increased aggression of wild-type males and *V1a2* ^*+/N68I*^ males (A, C). Injection of AVT did not increase aggression of *V1a2* ^*N68I/N68I*^ males (E). Injection of saline did not alter aggression of the wild-type males (B), *V1a2* ^*+/N68I*^ males (D), and *V1a2* ^*N68I/N68I*^ males (F). Mean ± SEM. Each n = 6, Wilcoxon signed-rank test: *P<0.05 VS before.
(TIFF)

S1 Table Summary of mutant phenotypes. (TIFF)

S1 Movie Mate-guarding behavior in the morning (1). The female has the largest abdomen. The near male approaches the female and interrupts the rival (the far male) from approaching the female. This movie is played at quadruple speed.
(MOV)

S2 Movie Mate-guarding behavior in the morning (2). The near male maintains its position between the female and the rival. This movie is played at quadruple speed.
(MOV)

S3 Movie Mate-guarding behavior before Manning Compound injection. The near male maintains its position between the female and the rival. This movie is played at quadruple speed.
(MOV)

S4 Movie Mate-guarding behavior of the near male 5-min after Manning Compound injection. The near male (Manning compound injected male) did not interrupt the rival (uninjected male) from approaching the female, although its velocity is normal. This movie is played at quadruple speed.
(MOV)

S5 Movie Mate-guarding behavior of *V1a2* ^*N68I/N68I*^ males. The mutant did not interrupt the rival from approaching the female. This movie is played at quadruple speed.
(MOV)
