# Supplementary figures and images for: An Essential Role of the Arginine Vasotocin System in Mate-Guarding Behaviors in Triadic Relationships of Medaka Fish (Oryzias latipes)
Source: PLoS Genet. 2015 Feb 26;11(2):e1005009. doi: 10.1371/journal.pgen.1005009 (PMC4342251; doi:10.1371/journal.pgen.1005009)

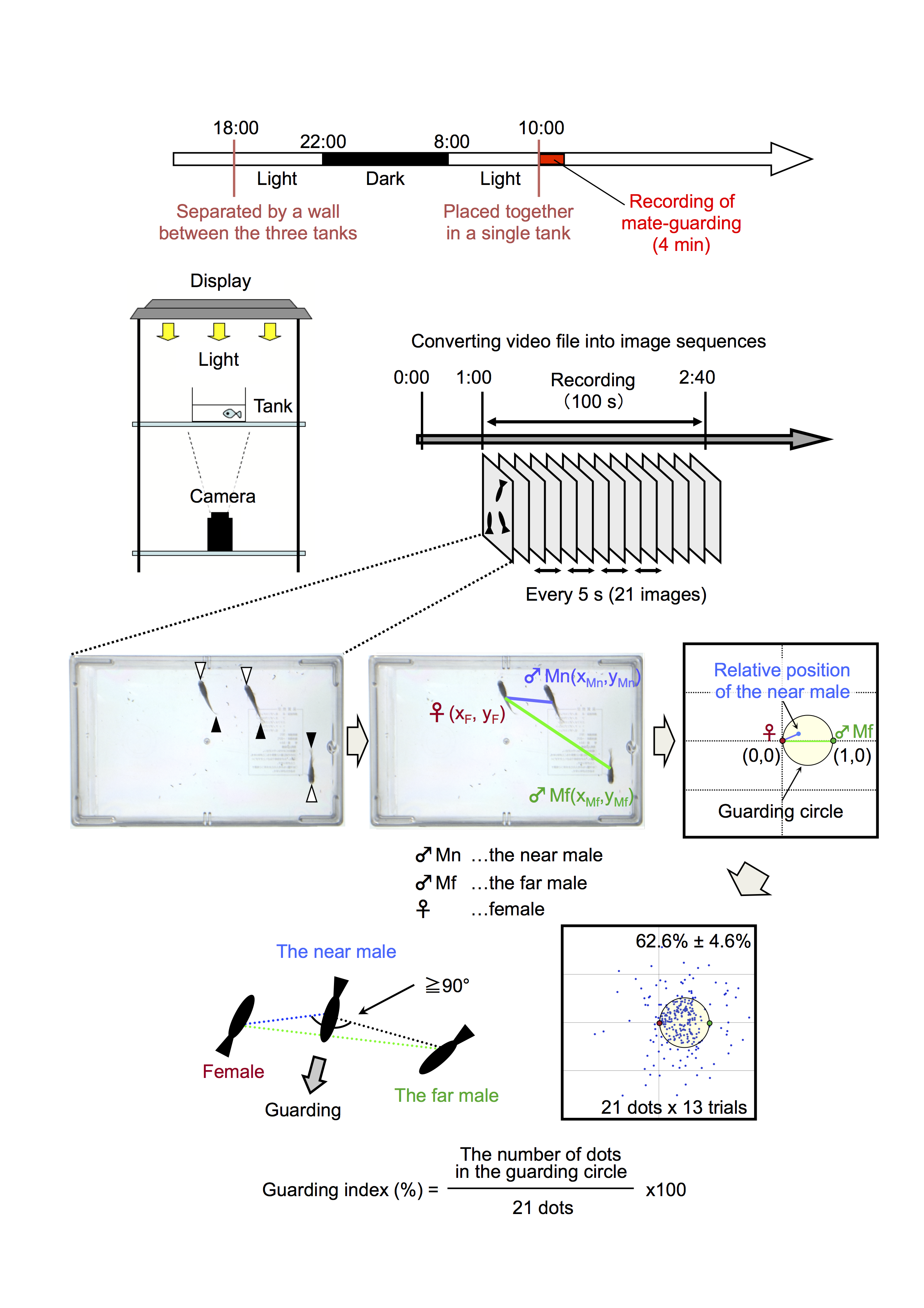

Supplement: S1 Fig — Upper left: Set-up for recording mate-guarding behavior. Fish movement was recorded from underneath, as the tank is transparent. Upper right: Time-course for the behavioral test. We separated the three fish before the behavioral test, and then put the three fish into the same tank for the behavioral measures to control the timing of spawning. We determined the time (~10:00) for the behavioral test following an established method for mating assay published in our previous study [15]. Lights were turned off at 22:00. We converted video files into 21 image sequences per 5 s, and manually measured the head and tail positions of the three medaka fish using ImageJ (NIH) to calculate the center positions, which were used as their body positions. The male with a shorter mean distance for 100 s from the female than the other male was defined as the “near male”, and the other male was defined as the “far male”. Based on the positions of the female (xF, yF), the far male (xMf, yMf), and the near male (xMn, yMn), the relative positions of the near male (X, Y) were calculated by the formula described in the text when the positions of the female and the far male were defined as (0, 0) and (1, 0), respectively. We spotted the relative positions of the near male and defined the “guarding circle” as a circle with center (1/2, 0) and radius 1/2. When the near male is present in the guarding circle, the near male remains near the female and interferes with the rival (the far male). Thus, we defined the probability of being in the guarding circle as an index representing the degree of mate-guarding (guarding index). (TIFF) [file pgen.1005009.s001.tiff]

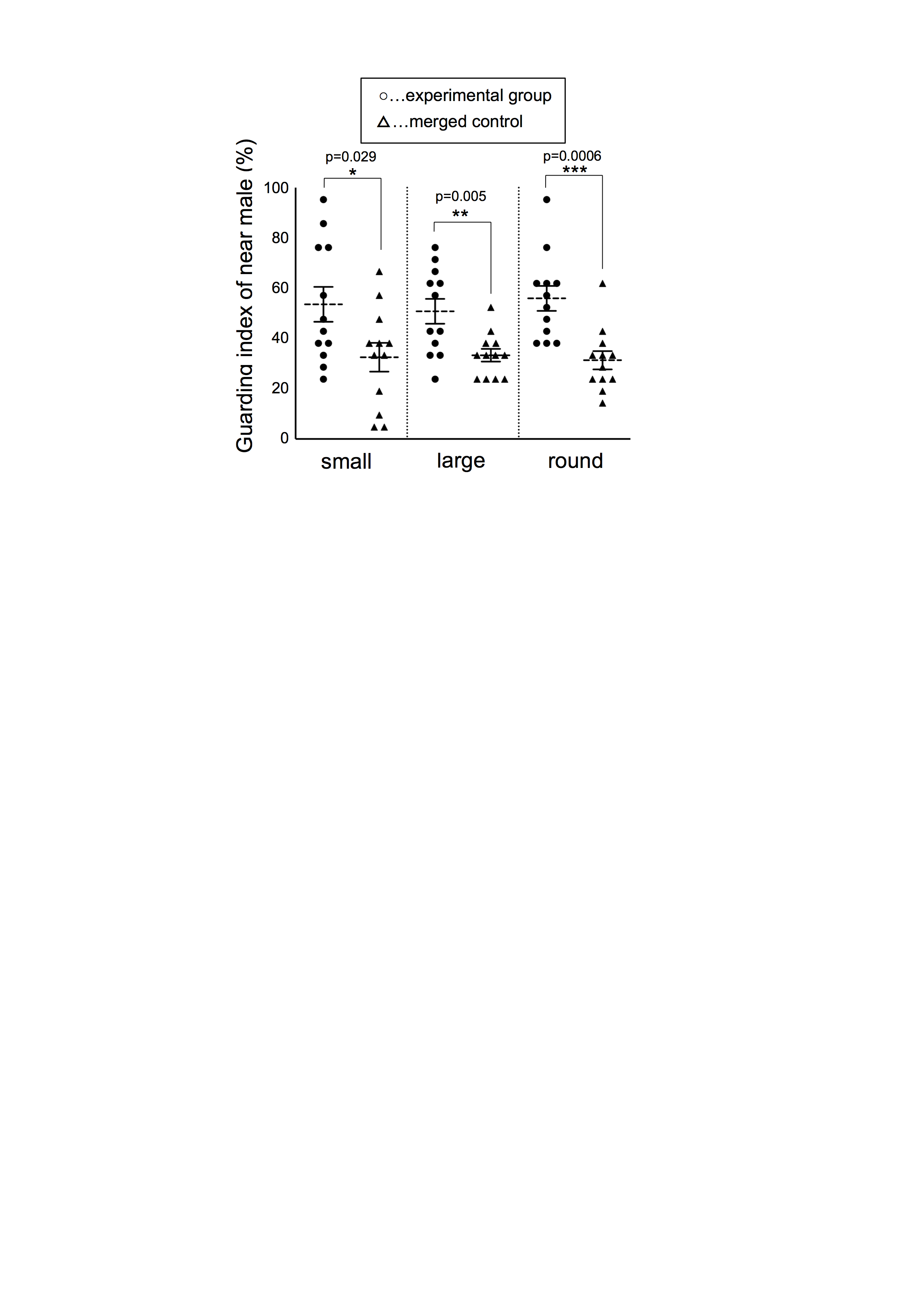

Supplement: S2 Fig — The size (small, 9.5 cm x 13 cm x 12 cm (height); large, 21 cm x 30 cm x 10 cm (height)) and shape (15-cm diameter circular tank) of the tank did not influence this behavior. Water depth was about 3–4 cm. Mean ± SEM. Each n = 12, Student’s t-test: *P<0.05, **P<0.01, ***P<0.001. (TIFF) [file pgen.1005009.s002.tiff]

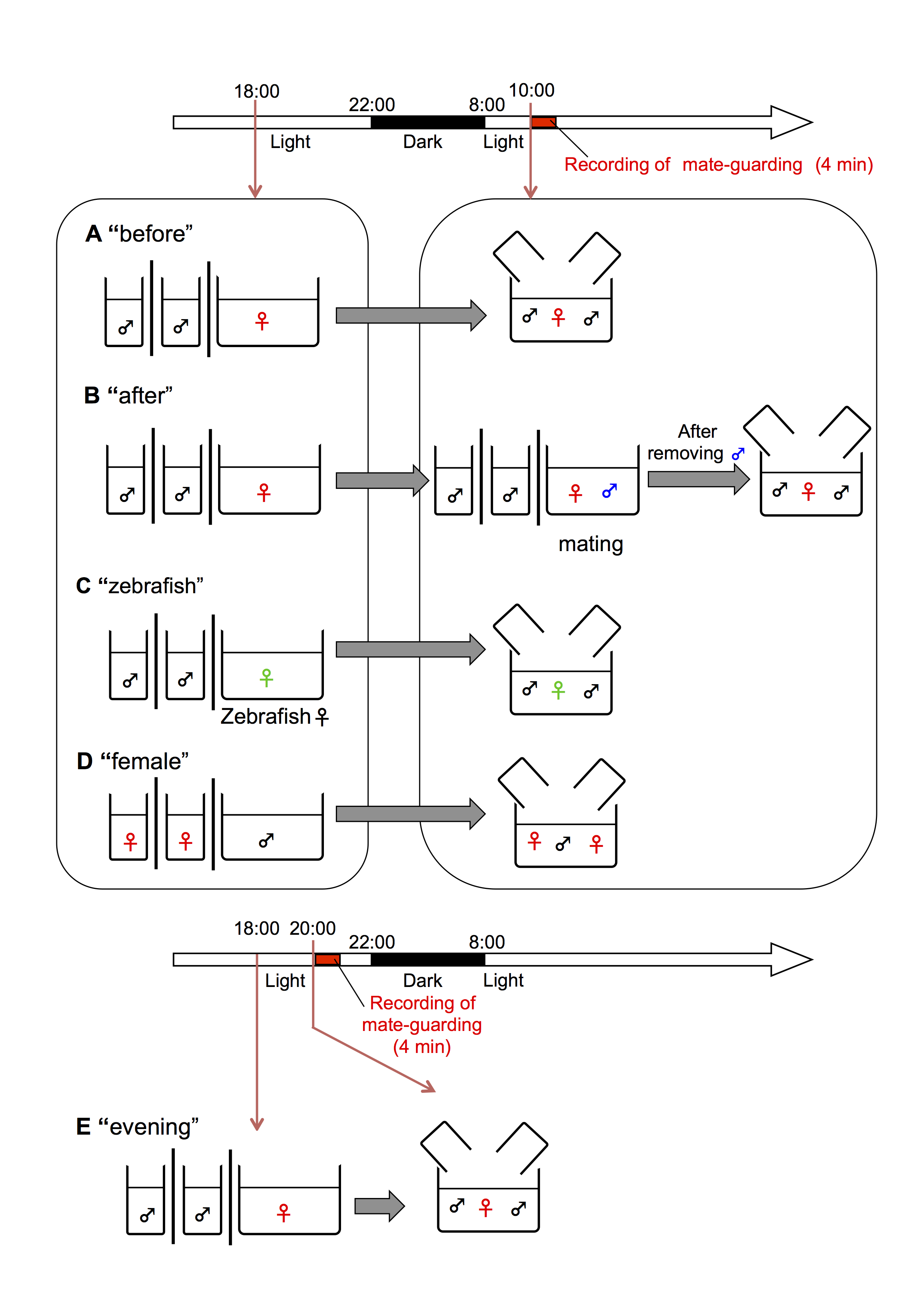

Supplement: S3 Fig — (A) “Before”: two males and a female were separated in the evening on the day before the assay and the next morning were placed together in a single tank. (B) “After”: two males and a female were separated in the evening on the day before the assay. The next morning, we allowed a third male, which was not used in the guarding test to mate with the female. After that, we removed the male for mating and placed two males (separated on the day before the assay) and one female together in a single tank. (C) “Zebrafish”: two medaka males and a zebrafish female were separated in the evening on the day before the assay and the next morning, were placed together in a single tank. (D) “Female”: two females and a male were separated in the evening on the day before the assay and the next morning were placed together in a single tank. (E) “Evening”: two males and a female were separated in the evening and after ~2 hours were placed together in a single tank (20:00–21:00). (TIFF) [file pgen.1005009.s003.tiff]

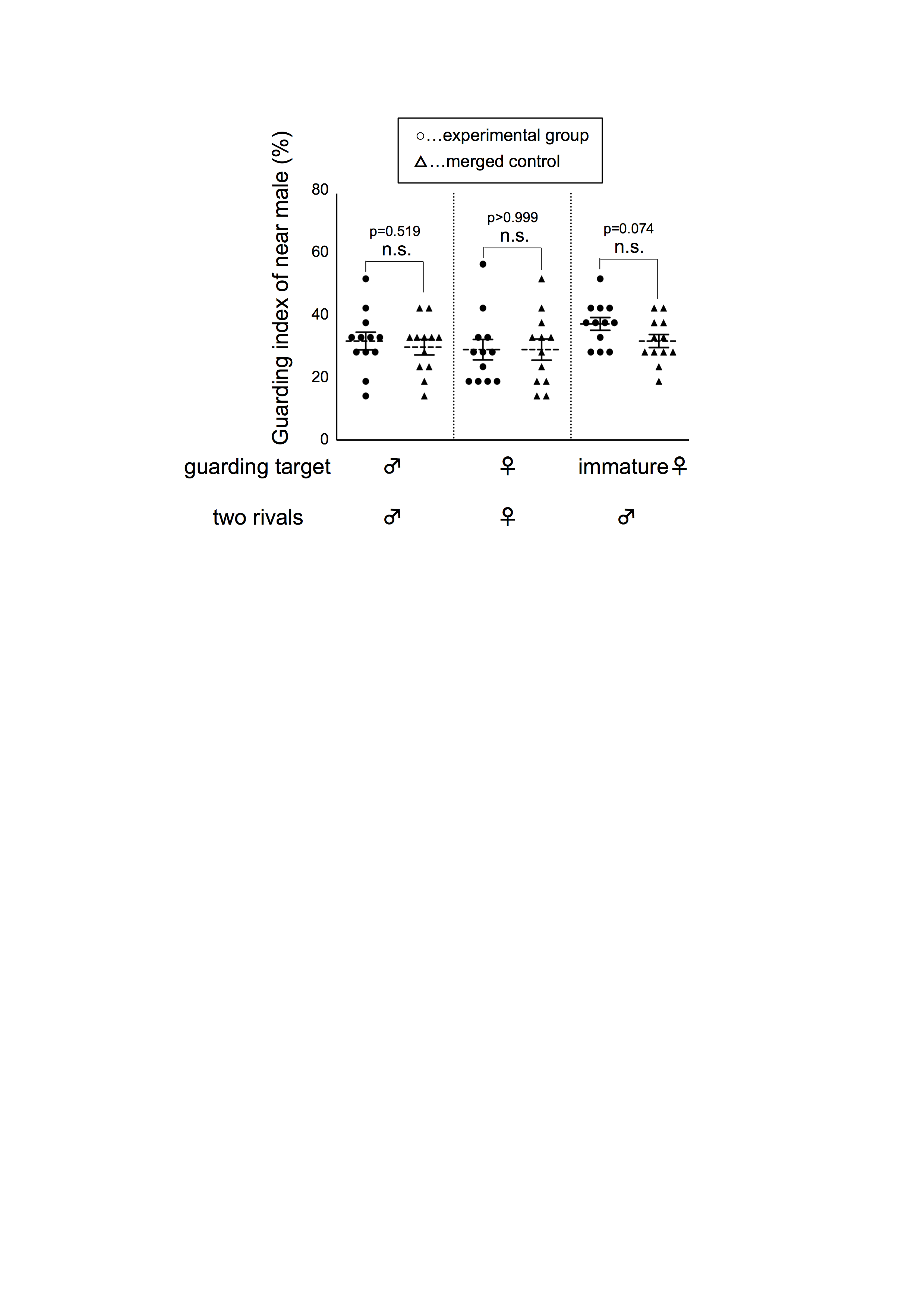

Supplement: S4 Fig — We used a male instead of a female as a guarding target (left), two females instead of two males as rivals (middle), and an immature female (about 2 months old, 2.1 cm-2.2 cm, 130 mg–140 mg) instead of a mature female (right) as a guarding target. In all the cases, two rivals did not exhibit mate-guarding toward a guarding target. Mean ± SEM. Each n = 12, Student’s t-test. (TIFF) [file pgen.1005009.s004.tiff]

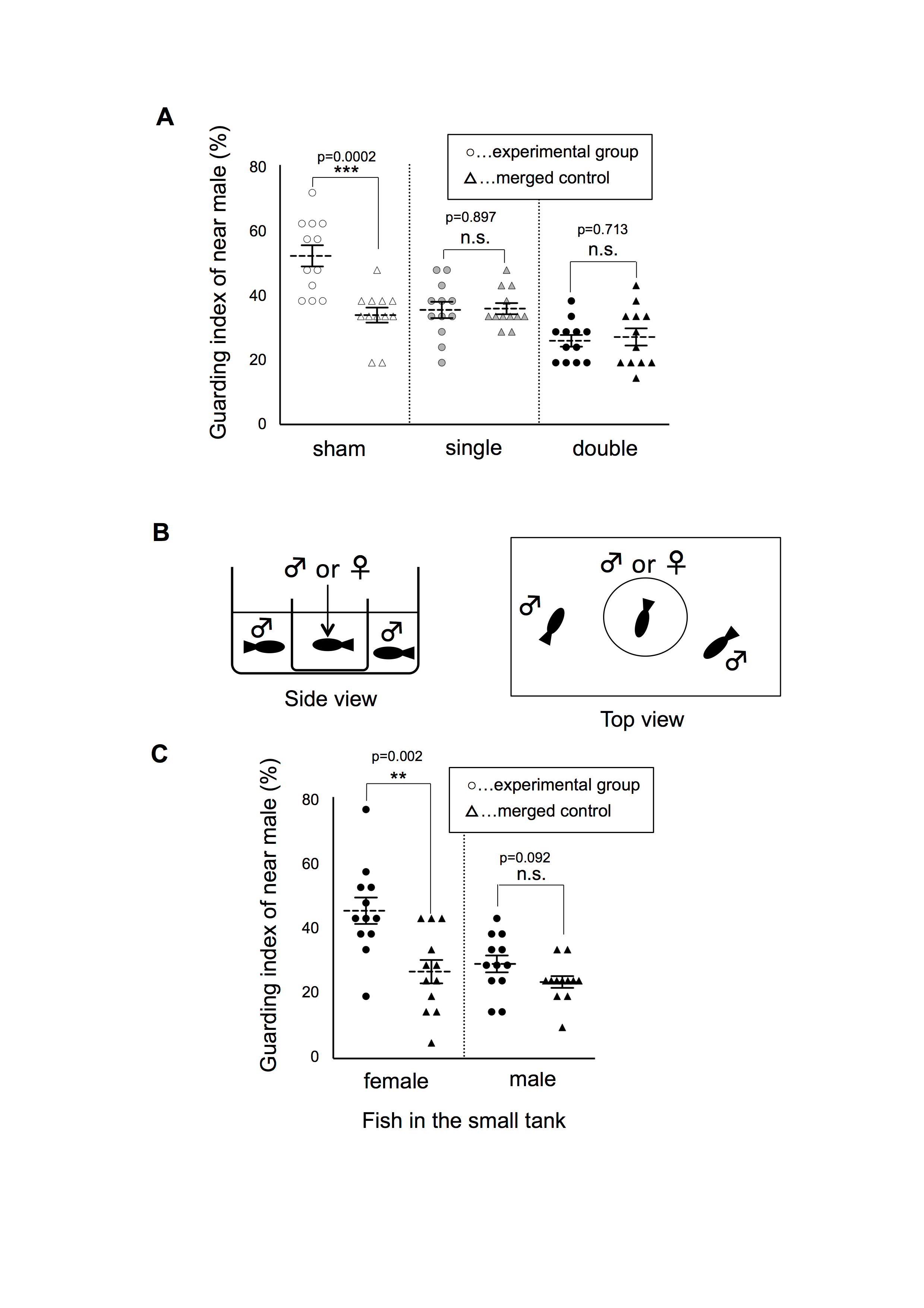

Supplement: S5 Fig — (A) Unilateral and bilateral eye-ablated males did not exhibit mate-guarding. Sham fish were injured just above the eyes. Single and Double: One or two eyes were removed, respectively. Mean ± SEM. Each n = 12, Student’s t-test: ***P<0.001. (B) Procedure for the male or female isolated mate-guarding experiments, we placed a small transparent tank (6 cm diameter circular tank) in the center of the test tank. A female or male was placed in the small circular tank and two other males were placed in outside of the small tank (the water in the outer tank did not contain pheromones of the fish in the small tank). (C) Visual information is sufficient for males to exhibit mate-guarding. Mean ± SEM. Each n = 12, Student’s t-test: **P<0.01. (TIFF) [file pgen.1005009.s005.tiff]

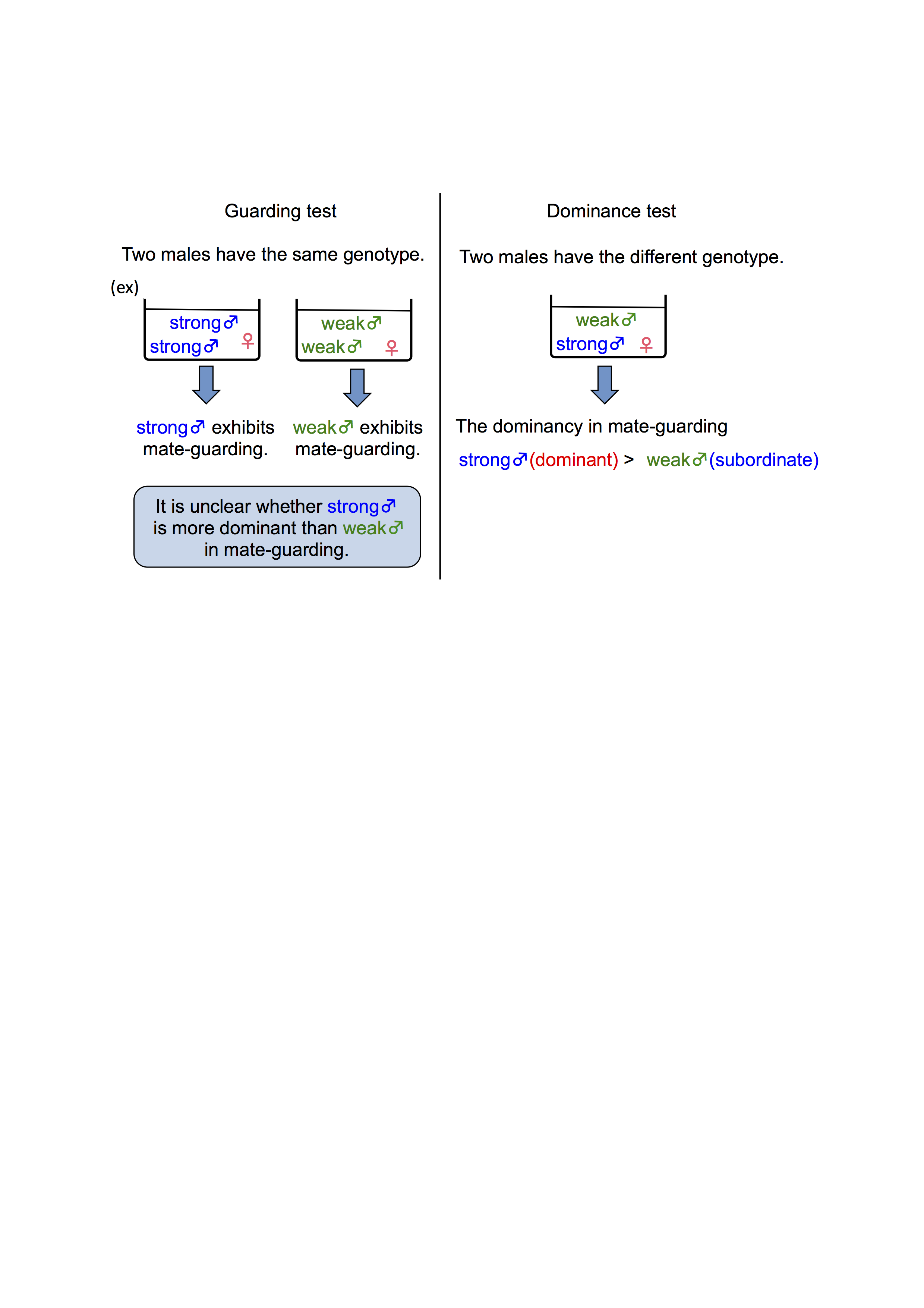

Supplement: S6 Fig — In the guarding test, we used two males with the same genotype, so we could judge whether or not the males exhibited mate-guarding behavior. In this test, however, the mate-guarding can emerge irrespective of the strength of used males, as the guarding indices are altered according the strength of the rival males. In the dominance test, we can directly compare the guarding indices between two different genotypes, because a triadic setup comprises two males with different genotypes. For example, in the guarding test using avt mutants (Fig. 3C), the guarding index of near males of the two weak males in the dominance test (avt homozygote mutants) was as high as that of the two strong males in the dominance test (avt heterozygote mutants), because the rival males were also weak in a triadic setup comprising the two weak males. Thus we cannot directly compare the guarding indices across different genotype based on guarding test. (TIFF) [file pgen.1005009.s006.tiff]

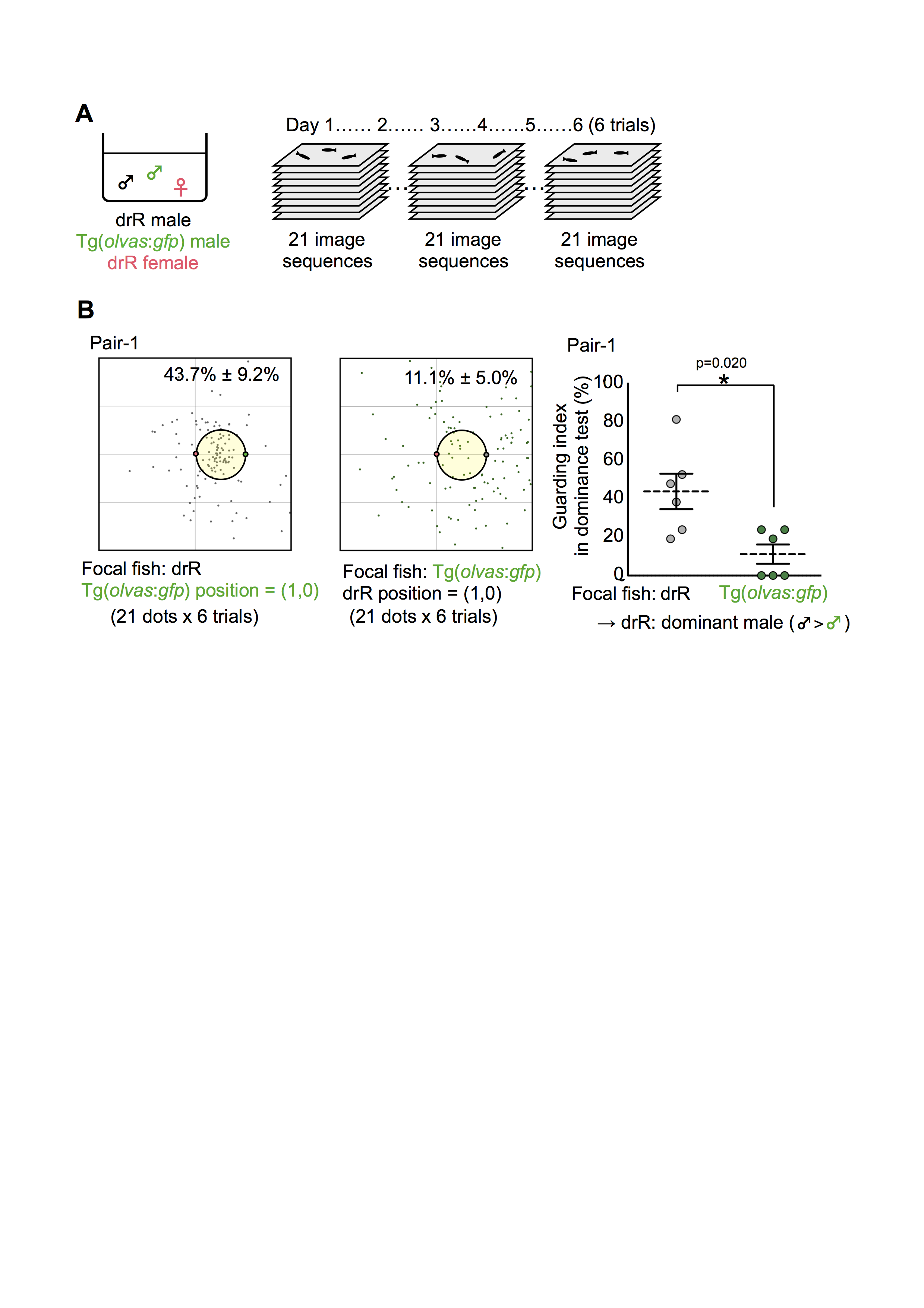

Supplement: S7 Fig — (A) We integrated the results of a 6-d dominance test and compared the guarding index of each genotype and judged which male was dominant by Mann-Whitney U test. (B) Pair-1 is shown as an example. The guarding index of the wild-type (drR) male (43.7%) was significantly higher than that of the transgenic (Tg; homozygote olvas:gfp) male (11.1%; Mann-Whitney U test, P = 0.020, n = 6). In this case, we judged that the wild-type (drR) male was dominant. If there was no significant difference (Mann-Whitney U test, P>0.05, n = 6), we judged that the two fish were equal. In Fig. 2C, 17 pairs were classified into three groups: the wild-type dominant pairs (n = 7, ♂ > ♂), Tg(homozygote olvas:gfp)-dominant pairs (n = 5, ♂ < ♂), and equivalent pairs (n = 5, ♂ = ♂). In Fig. 2C, we compared the percentage of GFP-positive eggs, indicating the Tg progeny rate, among the three groups. (TIFF) [file pgen.1005009.s007.tiff]

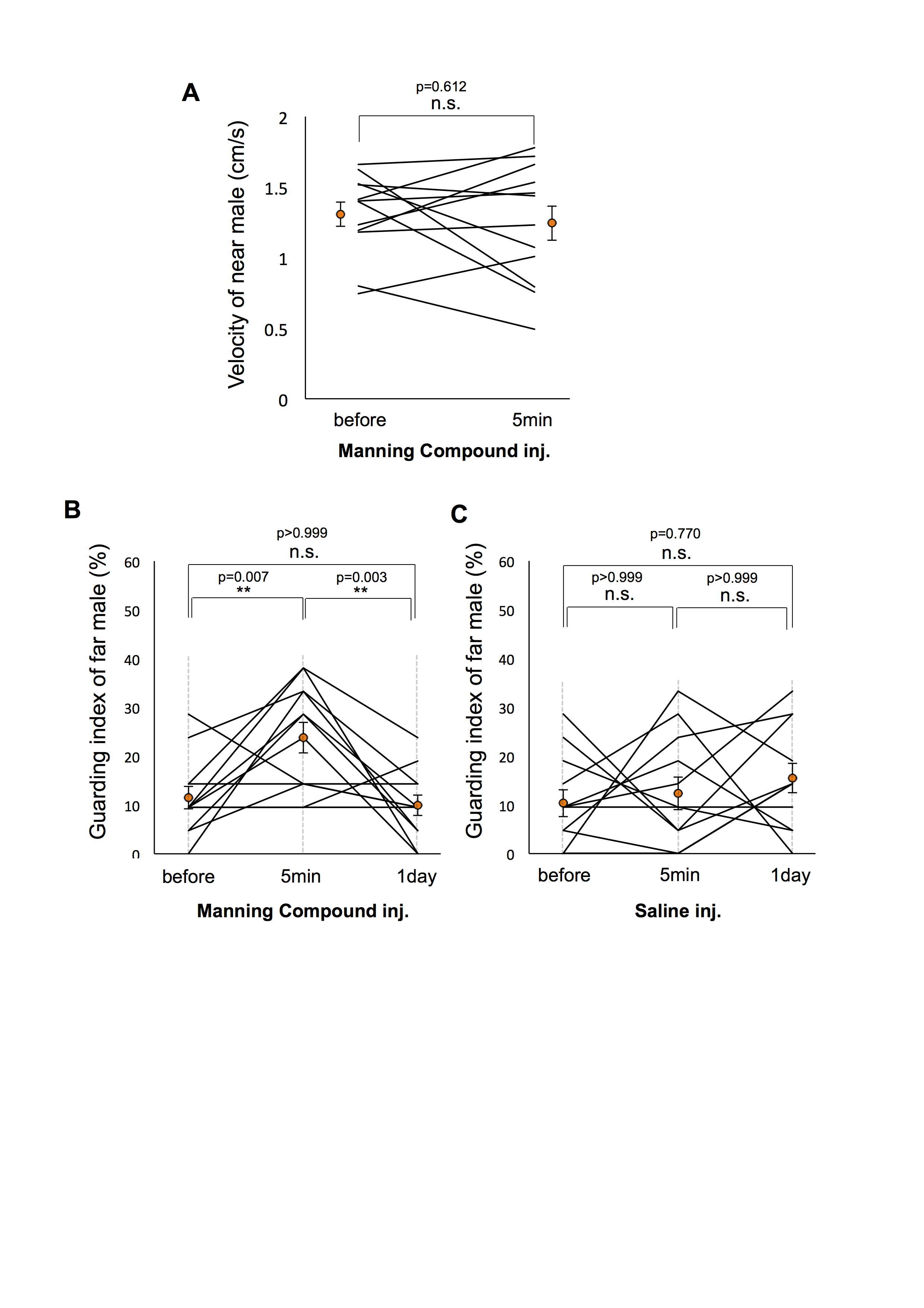

Supplement: S8 Fig — (A) The velocities of near males were not changed by injecting the Manning compound. Mean ± SEM. Each n = 12, Paired t-test. (B) The guarding indices of the far males (uninjected males) were significantly increased by injecting the Manning compound into the near males (5 min after injection) and this tendency disappeared 1 day after the injection. Mean ± SEM. Each n = 12, one-way repeated measures ANOVA with Bonferroni’s correction for multiple comparisons. **P<0.01 (C) The guarding indices of far males (uninjected males) were not altered by saline injection into near males. Mean ± SEM. Each n = 12, one-way repeated measures ANOVA with Bonferroni’s correction for multiple comparisons. (TIFF) [file pgen.1005009.s008.tiff]

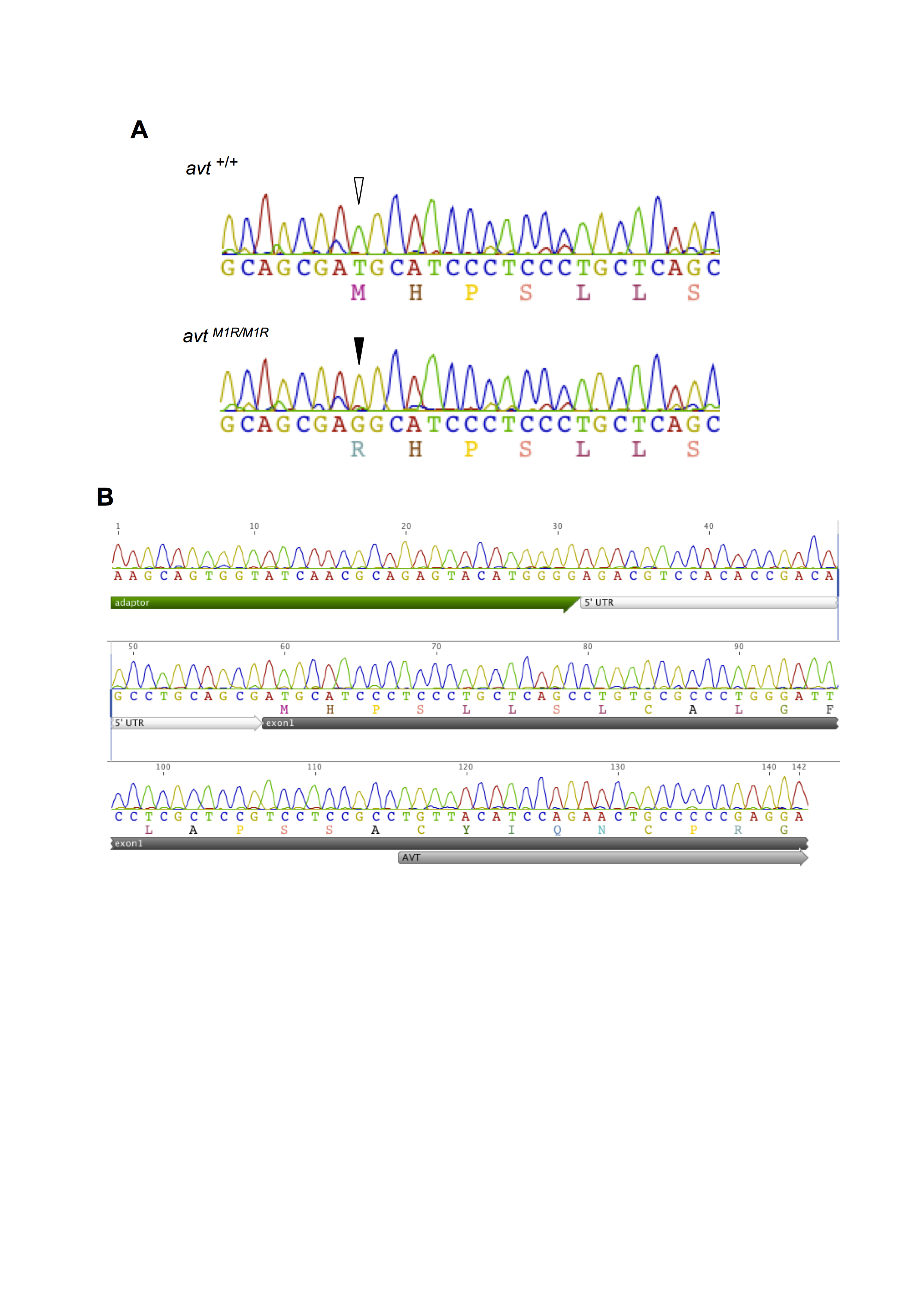

Supplement: S9 Fig — (A) A local sequence dataset comparing the wild-type avt (avt +/+) and avtM1R homozygotes (avt M1R/M1R) demonstrating the avt T2G mutation in avtM1R mutants (black arrowhead). (B) A local sequence of 5’-RACE product confirming the transcription initiation site of avt, which was predicted by the annotated avt sequence. We sequenced 10 and 9 cDNA clones derived from the drR and cab strains, respectively and confirmed that the sequences of all 19 cDNA clones started from the transcription initiation site, which was predicted by the annotated avt sequence. “adaptor”: added nucleotide in SMARTer RACE cDNA Amplification Kit (Clontech). (TIFF) [file pgen.1005009.s009.tiff]

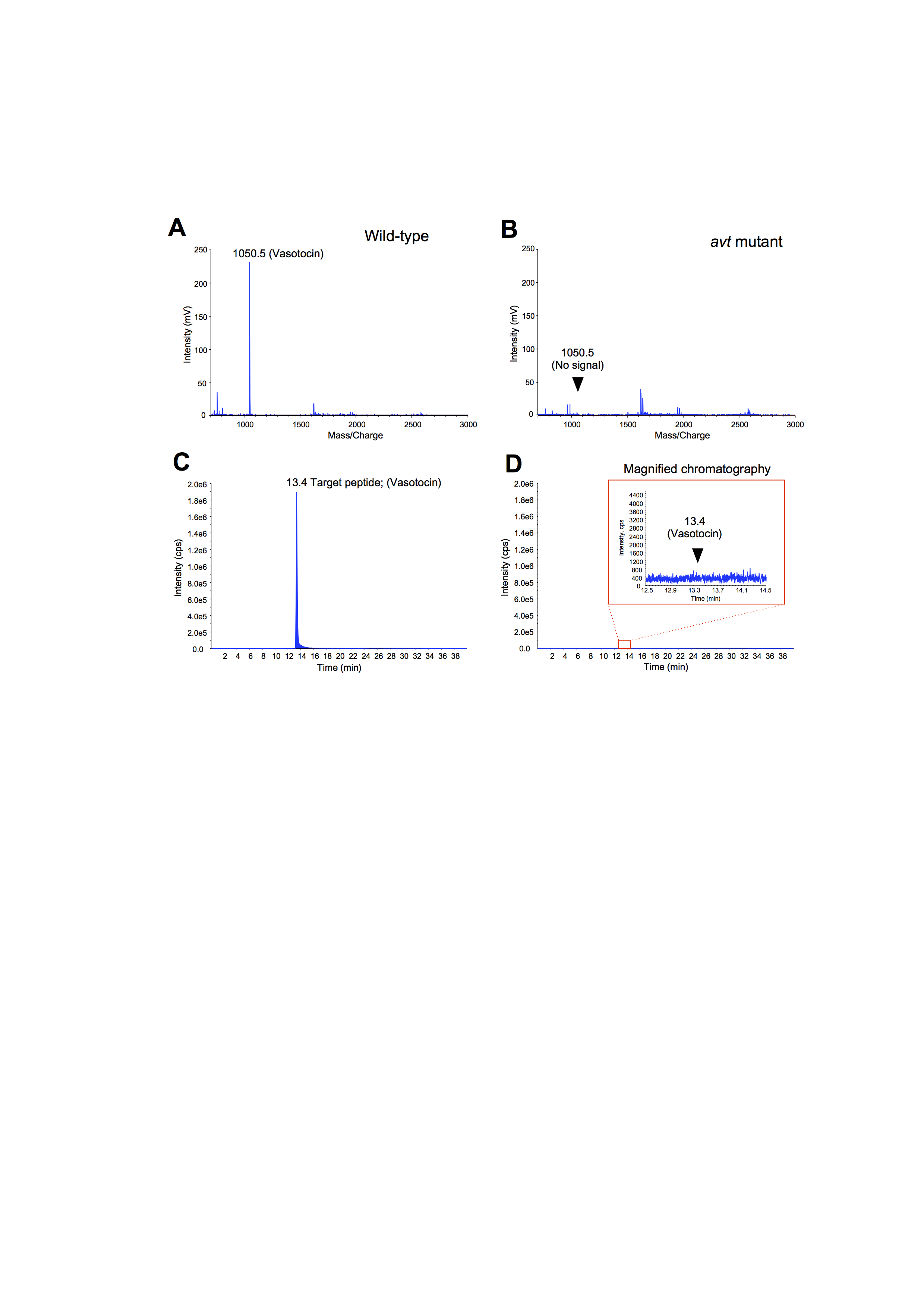

Supplement: S10 Fig — AVT peptides are present in the pituitary of the wild-type (Cab), but not in the avt mutant. MALDI-TOF MS spectra of the peptides from the pituitary in the wild-type (A) and avt mutant (B) brains. The x-axis shows the m/z, mass to charge ratio; the y-axis shows the intensity of the molecular ions. An ion peak at m/z 1050.5 indicated the presence of the AVT peptide in the wild-type (A). For the SRM assay, we selected Q1 (precursor ion: 525.8)/Q3 (fragment ion y3: 328.2), based on tandem MS spectrum of [Arg8]-vasotocin (Sigma Aldrich, V0130). Abundant AVT peptides were detected in the wild-type (C), while no AVT peptide was detected in avt mutant (D). The x-axis shows retention time; the y-axis shows the intensity of the molecular ions. (TIFF) [file pgen.1005009.s010.tiff]

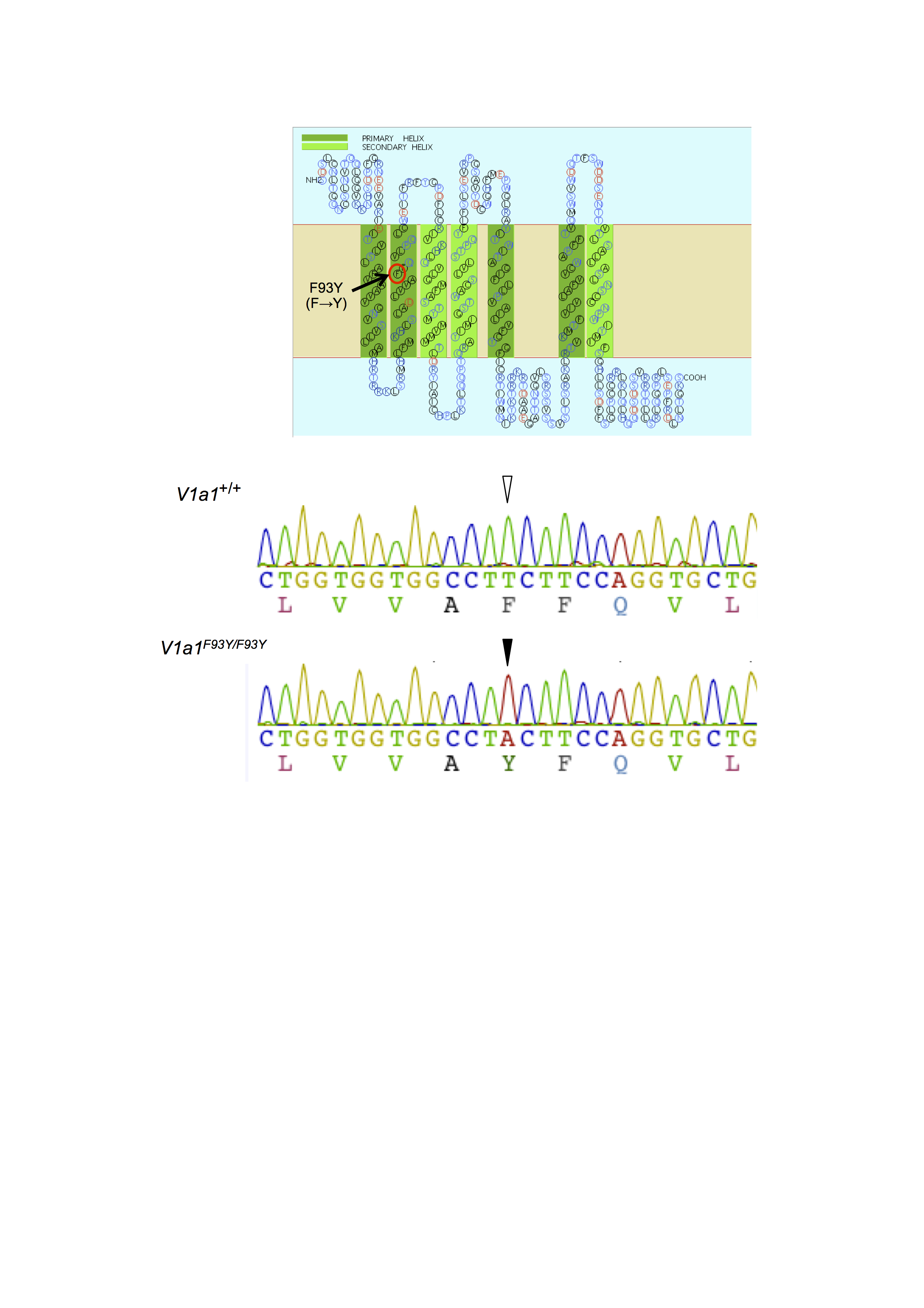

Supplement: S11 Fig — A local sequence dataset comparing the wild-type V1a1 (V1a1 +/+) and V1a1F93Y homozygotes (V1a1 F93Y/F93Y) demonstrating the V1a1 T278A mutation in V1a1F93Y mutants (black arrowhead). (TIFF) [file pgen.1005009.s011.tiff]

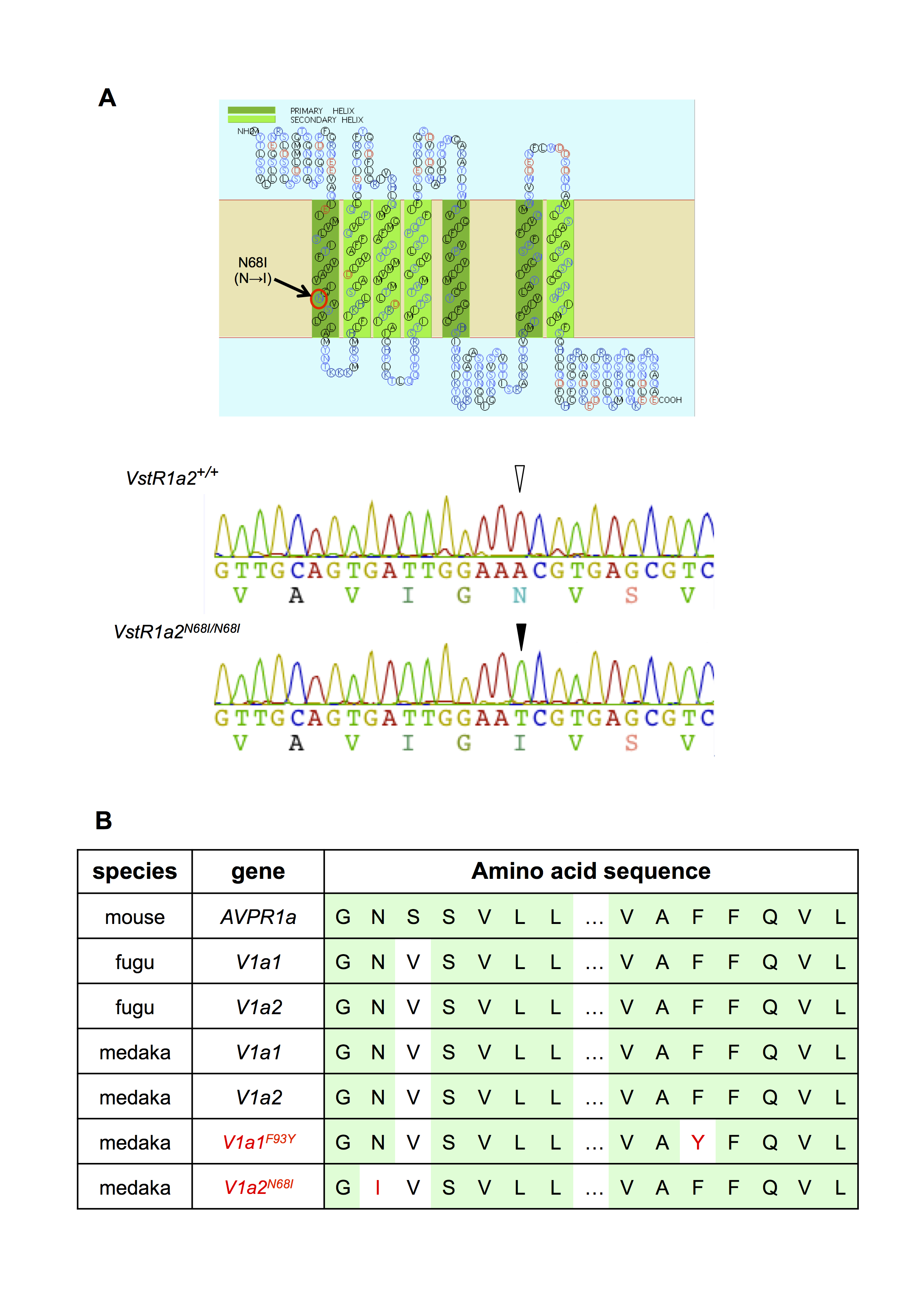

Supplement: S12 Fig — (A) A local sequence dataset comparing the wild-type V1a2 (V1a2 +/+) and V1a2N68I homozygotes (V1a2 N68I/N68I) demonstrating the V1a2 A203T mutation in V1a2N68I mutants (black arrowhead). (B) The primary structure of V1a receptor paralogs in mouse and fish. Phenylalanine 93 and arginine 68, which are identical among known forms, were changed to tyrosine and isoleucine in V1a1F93Y and V1a2N68I mutant alleles, respectively (red letters). (TIFF) [file pgen.1005009.s012.tiff]

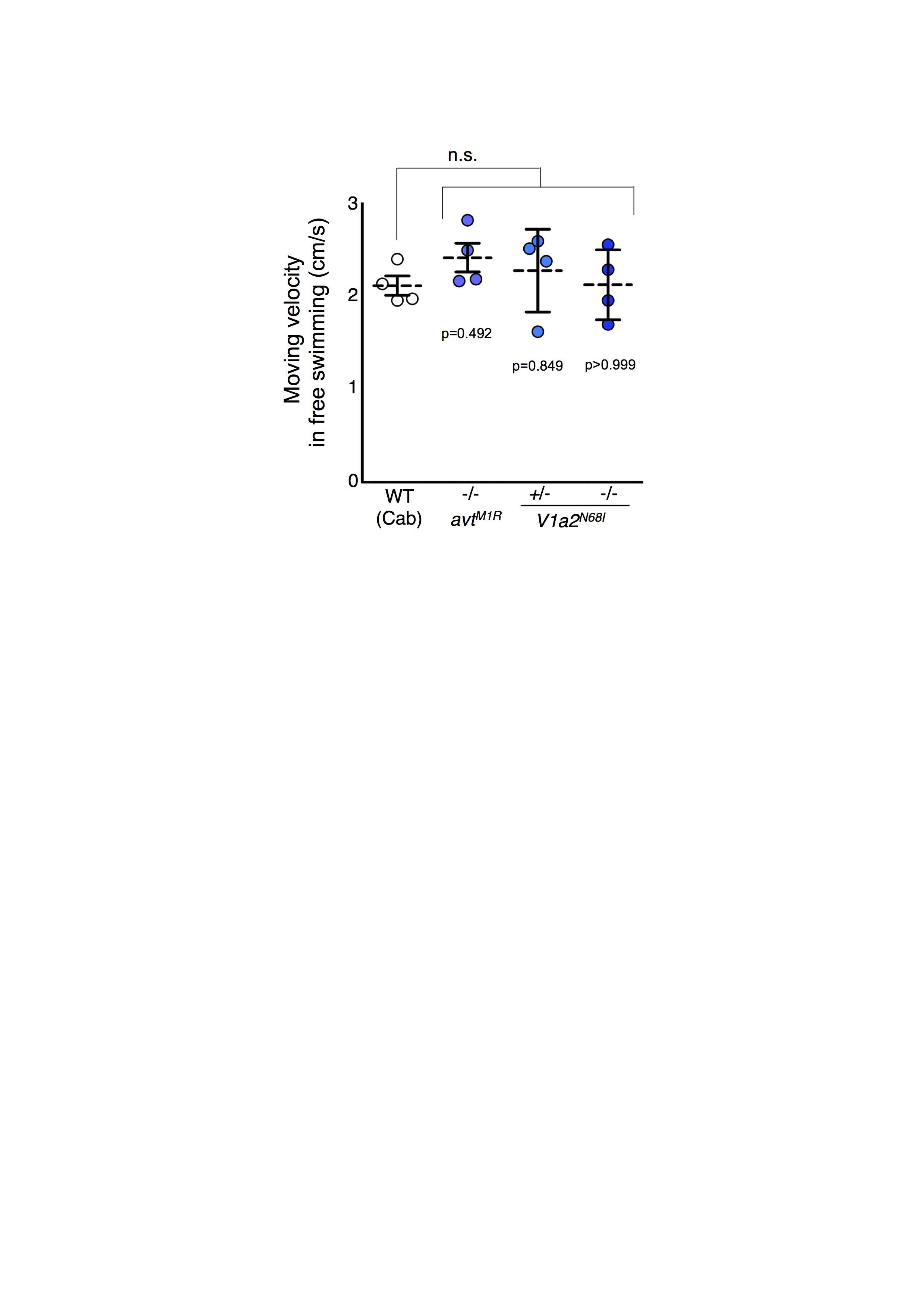

Supplement: S13 Fig — One minute after we placed 1 fish in the tank, we calculated its movement velocity for 60 s. Mean ± SEM. Each n = 4, Dunnett’s test. (TIFF) [file pgen.1005009.s013.tiff]

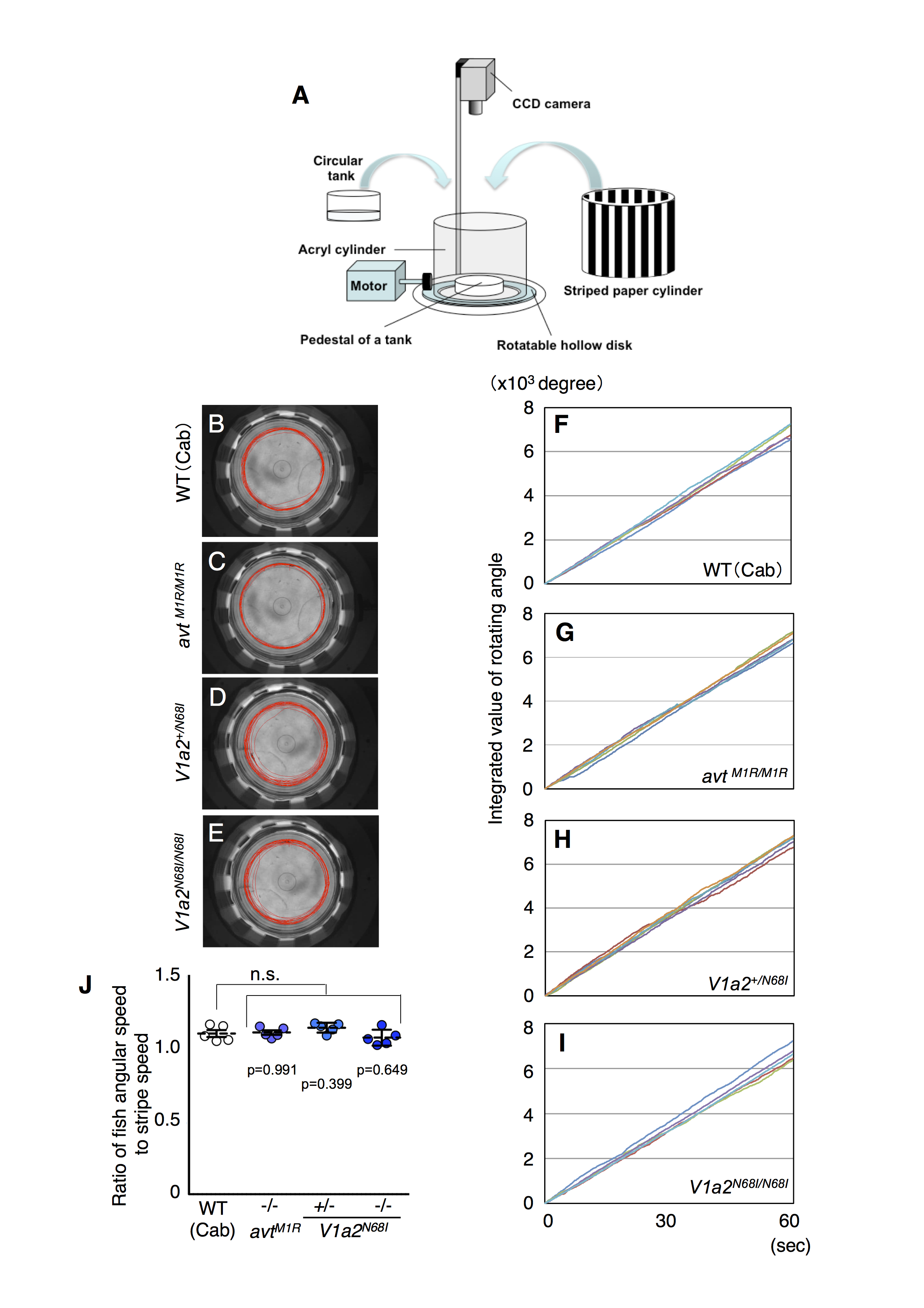

Supplement: S14 Fig — (A) Equipment for the analysis of the OMR described previously [65]. (B-E) Representative examples of traces of fish movement during OMR. (F-I) Integrated angular velocity during 60 s of (F) wild-type (Cab), (G) avt M1R/M1R, (H) V1a2 +/N68I, and (I) V1a2 N68I/N68I fish. Each line indicates integrated angular velocity of five individual fish. (J) Ratio of the mean fish angular speed to that of the stripe speed. Mean ± SEM. Each n = 5, Dunnett’s test. (TIFF) [file pgen.1005009.s014.tiff]

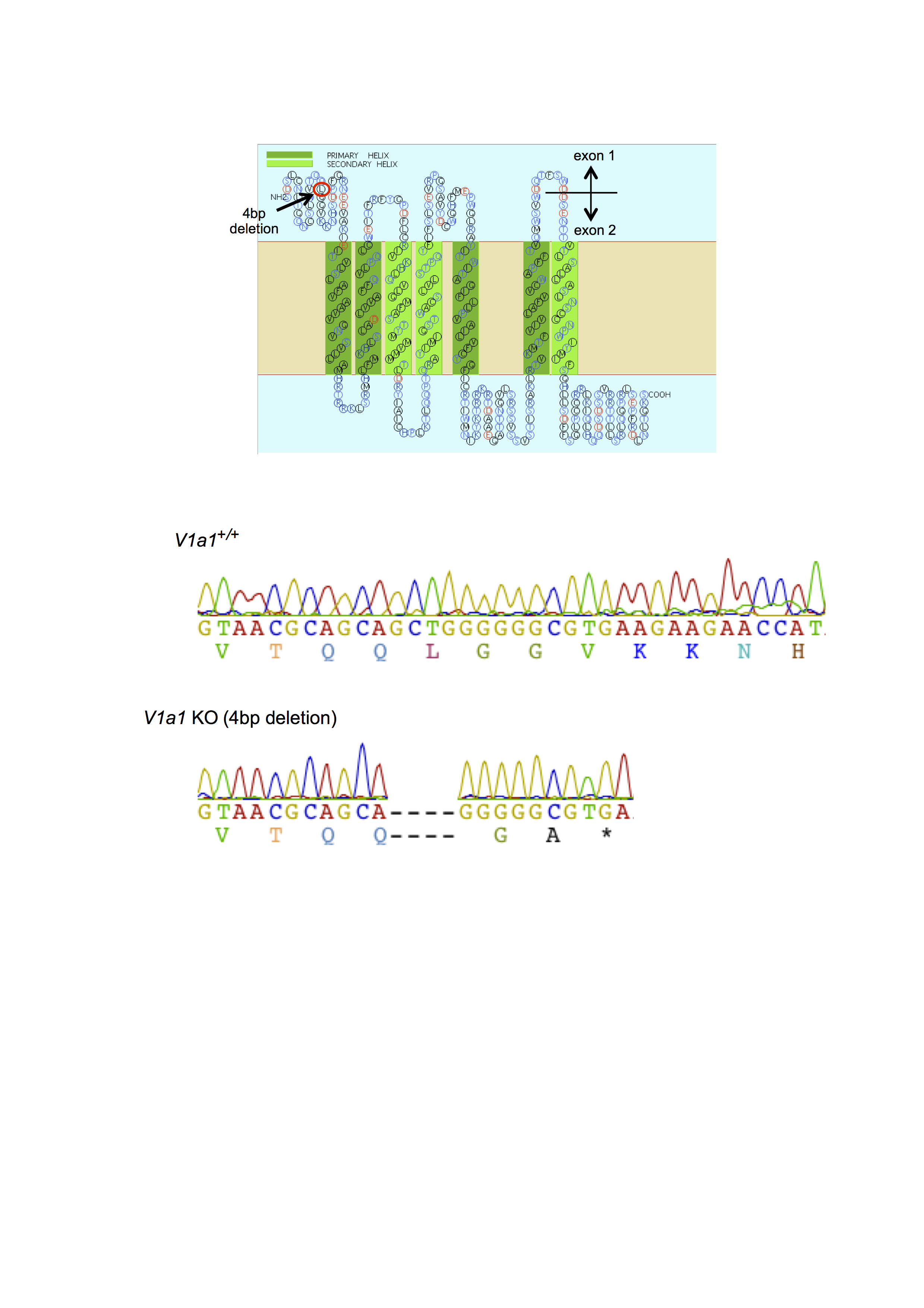

Supplement: S15 Fig — A local sequence dataset comparing wild-type V1a1 (V1a1 +/+) and V1a1 knockout (KO) homozygotes demonstrating that a 4-bp deletion generated a nonsense mutation (G26X) in V1a1 KO mutants. V1a1 gene consists of two exons. The deletion was located in the first exon and the mutated transcripts encode C-terminal deleted proteins lacked six of the seven transmembrane domains encoded by the first exon. (TIFF) [file pgen.1005009.s015.tiff]

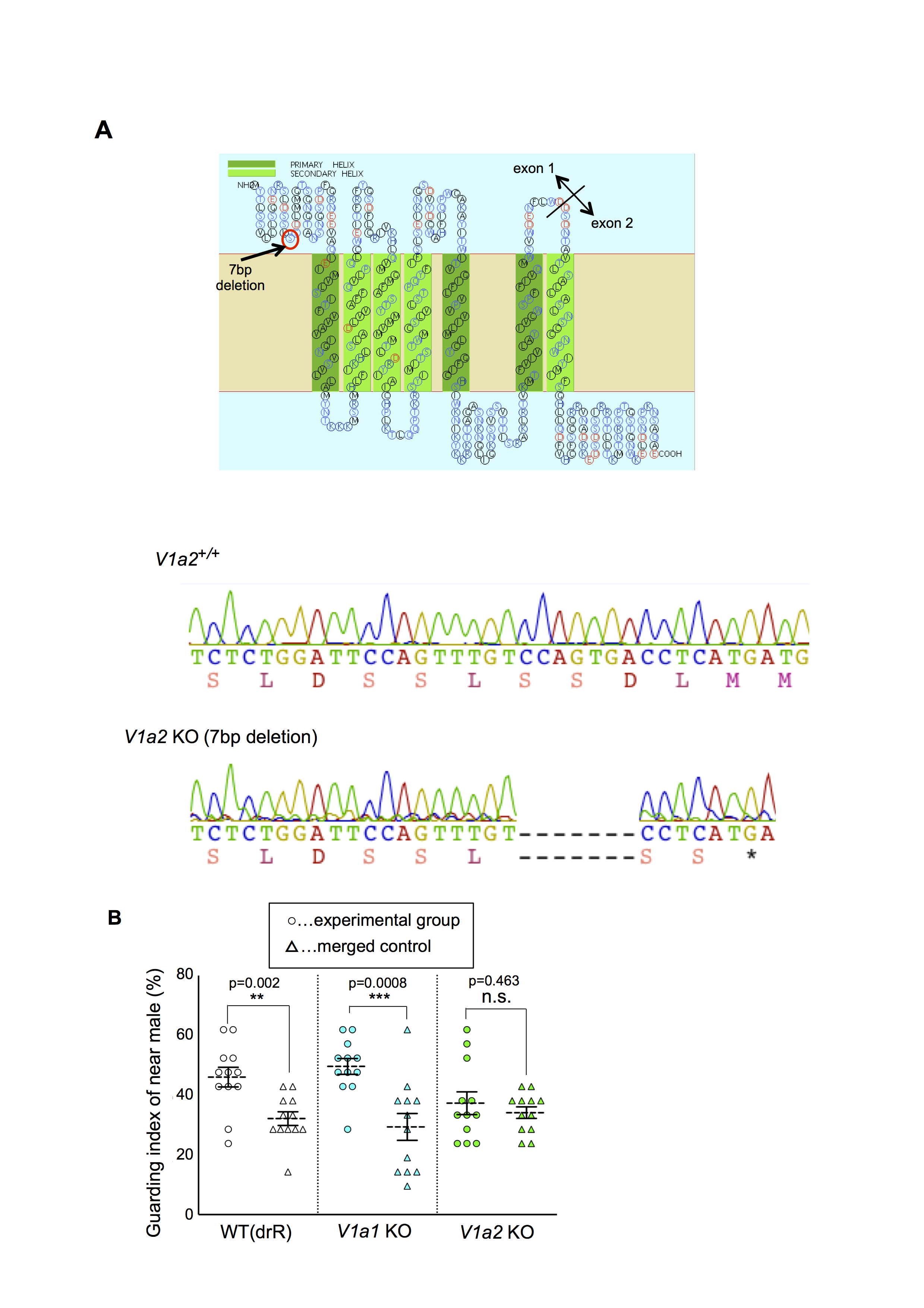

Supplement: S16 Fig — (A) A local sequence dataset comparing the wild-type V1a2 (V1a2 +/+) and V1a2 knockout (KO) homozygotes demonstrating that a 7-bp deletion generated a nonsense mutation (D24X) in V1a2 KO mutants. V1a2 gene consists of two exons. The deletion was located in the first exon and the mutated transcripts encode C-terminal deleted proteins lacked six of the seven transmembrane domains encoded by the first exon. (B) V1a1 KO mutants exhibited mate-guarding behavior, whereas V1a2 KO mutants did not. Mean ± SEM. Each n = 12, Student’s t-test: **P<0.01, ***P<0.001. (TIFF) [file pgen.1005009.s016.tiff]

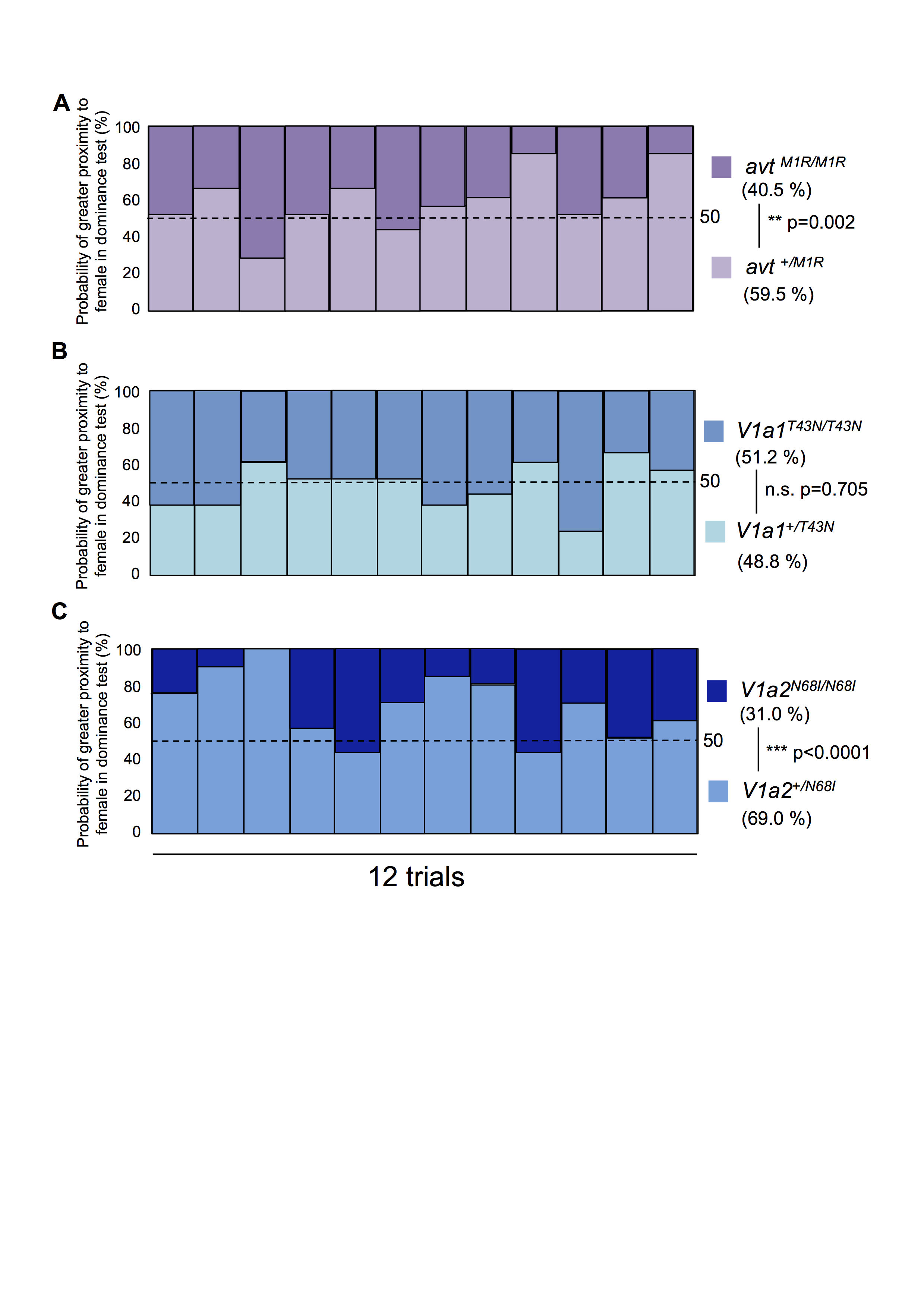

Supplement: S17 Fig — We judged which male was closer to the female in a total 256 images (21 images x 12 trials) in the dominance test (Fig. 3D). Each trial contained 21 images (5-s interval for 100 s. See S1 Fig.). Here we calculated the probabilities of being closer to the female between heterozygote and homozygote mutants based on the 256 images. We then detected a significant bias between the two probabilities using the chi-square test in avt (A) and V1a2 mutants (C), but not in V1a1 mutants (B). (TIFF) [file pgen.1005009.s017.tiff]

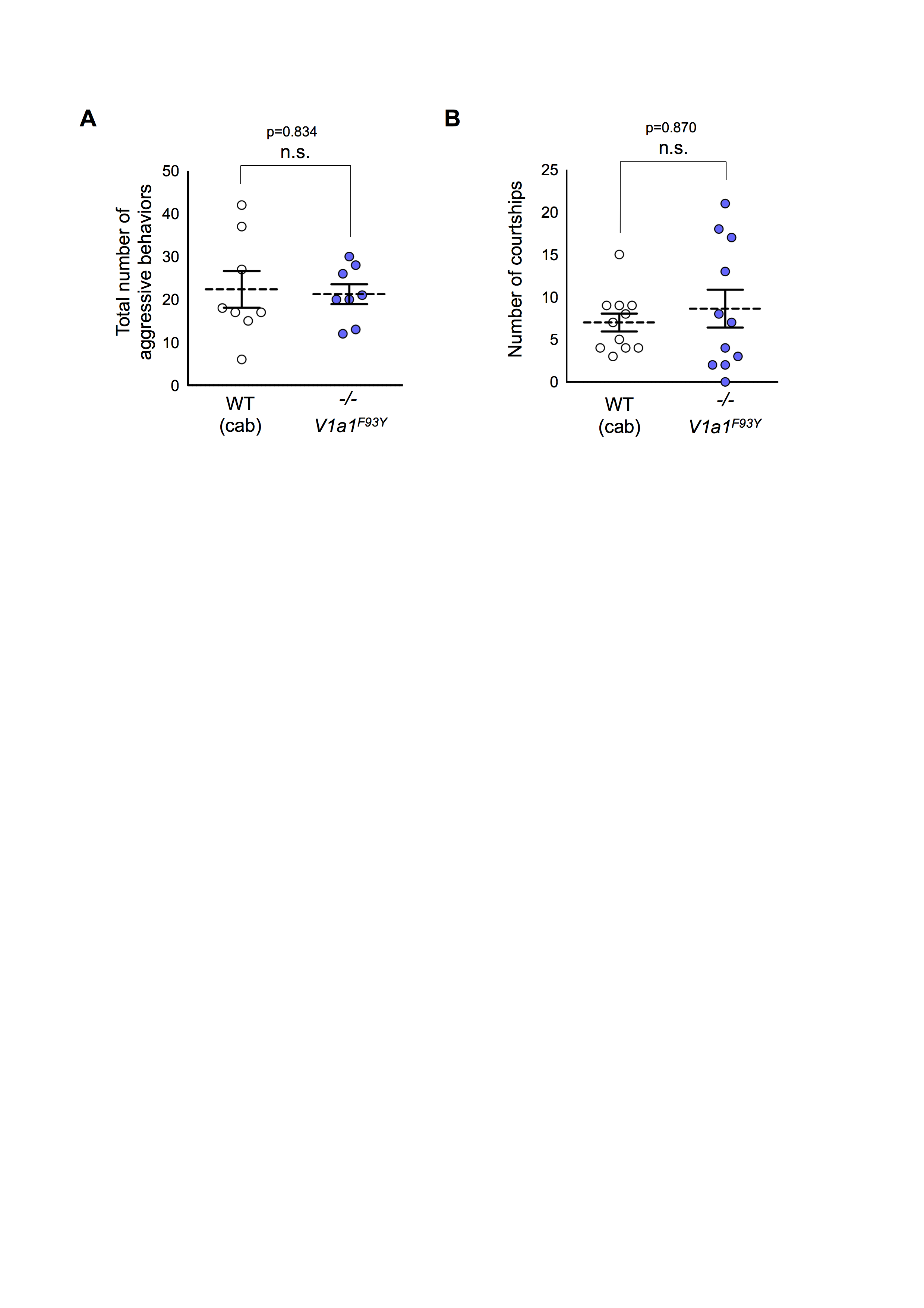

Supplement: S18 Fig — V1a1 F93Y homozygote mutant males exhibited normal aggressive behavior (A) and courtship behavior (B). Mean ± SEM. Each n = 8 (A), n = 11 (B), Mann-Whitney U-test. (TIFF) [file pgen.1005009.s018.tiff]

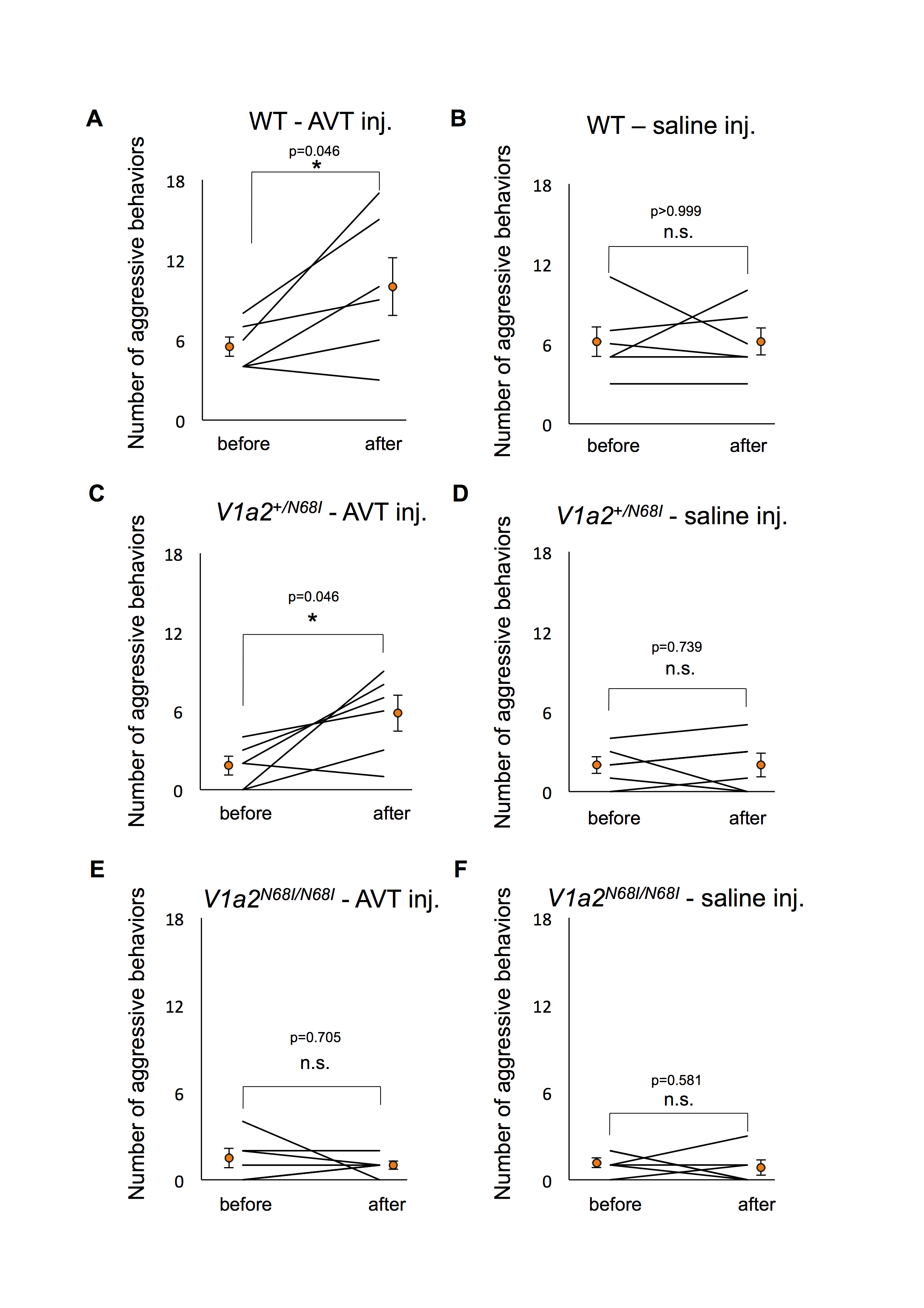

Supplement: S19 Fig — AVT or saline was injected to the one of three fish intraperitoneally and the number of aggressive behaviors of focal male toward other males before and after injection was counted. (A, C, E) Injection of AVT increased aggression of wild-type males and V1a2 +/N68I males (A, C). Injection of AVT did not increase aggression of V1a2 N68I/N68I males (E). Injection of saline did not alter aggression of the wild-type males (B), V1a2 +/N68I males (D), and V1a2 N68I/N68I males (F). Mean ± SEM. Each n = 6, Wilcoxon signed-rank test: *P<0.05 VS before. (TIFF) [file pgen.1005009.s019.tiff]

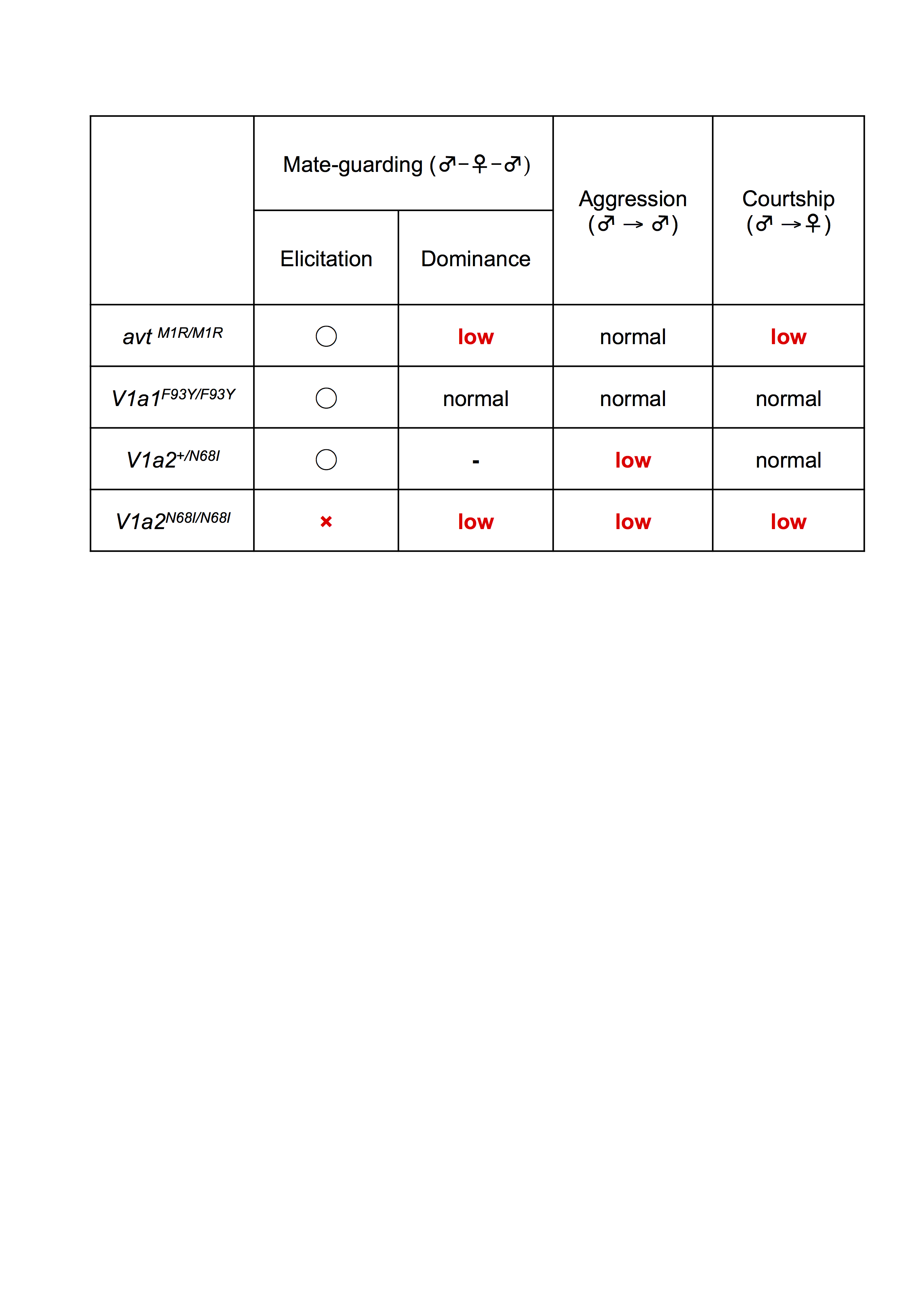

Supplement: S1 Table — (TIFF) [file pgen.1005009.s020.tiff]
